# Deciphering the spatiotemporal transcriptional and chromatin accessibility of human retinal organoid development at the single-cell level

**DOI:** 10.1016/j.isci.2024.109397

**Published:** 2024-03-04

**Authors:** Birthe Dorgau, Joseph Collin, Agata Rozanska, Veronika Boczonadi, Marina Moya-Molina, Adrienne Unsworth, Rafiqul Hussain, Jonathan Coxhead, Tamil Dhanaseelan, Lyle Armstrong, Rachel Queen, Majlinda Lako

**Affiliations:** 1 Biosciences Institute, Newcastle University, Newcastle upon Tyne NE1 3BZ, UK; 2 Newcells Biotech, Newcastle upon Tyne NE4 5BX, UK

**Keywords:** Genetics, Molecular biology, Neuroscience, Cell biology, Omics

## Abstract

Molecular information on the early stages of human retinal development remains scarce due to limitations in obtaining early human eye samples. Pluripotent stem cell-derived retinal organoids (ROs) provide an unprecedented opportunity for studying early retinogenesis. Using a combination of single cell RNA-seq and spatial transcriptomics we present for the first-time a single cell spatiotemporal transcriptome of RO development. Our data demonstrate that ROs recapitulate key events of retinogenesis including optic vesicle/cup formation, presence of a putative ciliary margin zone, emergence of retinal progenitor cells and their orderly differentiation to retinal neurons. Combining the scRNA- with scATAC-seq data, we were able to reveal cell-type specific transcription factor binding motifs on accessible chromatin at each stage of organoid development, and to show that chromatin accessibility is highly correlated to the developing human retina, but with some differences in the temporal emergence and abundance of some of the retinal neurons.

## Highlights

• Single cell analyses reveal putative ciliary margin (pCM) presence in retinal organoids
• PCM harbors early RPCs which differentiate to late RPCs and retinal neurons
• Single cell ATAC-seq data reveal novel TF binding motifs in RPCs and retinal neurons
• RO development largely recapitulates retinogenesis

## Graphical abstract

**Figure:**
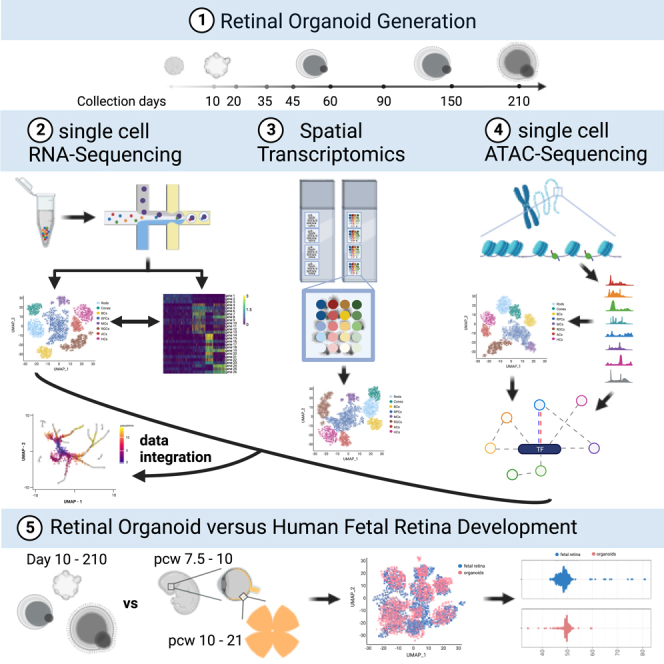

## Introduction

Retinal organoids (ROs) generated from human pluripotent stem cells (PSCs) provide a powerful platform for studying retinal development, disease modeling, and cell-based replacement therapies.^1^ Since their inception in early 2010s, multiple protocols have been developed by our group and others,^2^^,^^3^^,^^4^^,^^5^ resulting in laminated ROs with an apical putative outer nuclear layer packed full of photoreceptors, an inner nuclear layer comprising the retinal interneurons and a basal layer where retinal ganglion cells (RGCs) reside. Improvements in culture methods have led to the formation of a dense brush border comprising photoreceptor inner and outer-like segments,^6^ which respond to light in a manner similar to postnatal mice at the eye-opening stage.^7^^,^^8^^,^^9^ Single cell (sc) RNA-seq and immunofluorescence analyses have shown the presence of all the retinal cell types within the organoids, which transcriptionally convergence to adult peripheral retinal cell types.^8^^,^^10^ While the development rate of organoids *in vitro* and fetal retina *in vivo* is similar, the molecular and cellular composition of the organoids is highly dependent on the differentiation protocol.^11^

The dynamics of chromatin accessibility plays a critical role in regulating cell fate specification and maturation during development. The assay for transposase-accessible chromatin with high throughput sequencing (ATAC-Seq) has been employed to reveal the chromatin accessibility of PSC-derived ROs. Notably these studies have shown that chromatin accessibility dynamics recapitulates human retinogenesis, but some divergent features were also identified.^12^ For example, the accessible chromatin region near the transcription factor Ptf1a, which determines horizontal and amacrine cell fates during retinal development,^13^ is different between fetal retina and ROs, which could be in part responsible for the reduction in amacrine cells in the latter.^14^ Equally, an *RHO* enhancer, which is present in mature human rods, is missing in the RO rods:^15^ this could be the determining factor for the more modest expression of RHO in the organoids compared to human retina.

Spatial localization and cellular interactions are key determinants of cell fate and behavior. In ROs retinal lamination and positioning of cell types in a manner that allows functional connectivity is essential to recapitulate the physiological functions of adult retina. Current single cell studies to date lack single cell spatiotemporal resolution and thus it has not been possible to visualize the very rapid changes that characterize transitions from optic vesicle stage to optic cup or the emergence of the retinal neuroepithelium. While immunofluorescence studies indicate retinal progenitor cell (RPC) localization within the apical layer of ∼1 month old organoids,^16^ we are unsure where RPCs and neurogenic progenitors derived therefrom are first localized and whether a putative ciliary margin (pCM) is present in ROs. To fill this gap, we have employed for the first-time spatial transcriptomics (ST) in combination with scRNA- and ATAC-seq analyses in a time series of ROs comprising 8 time points from day 10 to day 210 of differentiation. This integrated analysis has enabled us to reconstruct the single cell spatiotemporal transcriptional and chromatin accessibility of ROs development, revealing the precise order of retinal neurons differentiation and their localization, which closely mimics human retinogenesis, but with some differences in the temporal emergence and abundance of some of the retinal cell types. Our data provide compelling evidence for the presence of pCM at the very edge of ROs, with potential to generate early RPCs. Our work provides the first integrated molecular and spatial atlas of human ROs development that could be used to identify novel genes and key pathways that underpin human retinal development and function.

## Results

### Single cell spatiotemporal phenotyping of ROs development

Eye-field specification, optic cup formation, and RPCs emergence present important milestones for human retinal development, however, these events are often difficult to study due to scarcity of human embryonic material. ROs derived from human PSCs have been shown to recapitulate the early retinal development^17^ and were used herein to generate a single cell spatiotemporal atlas up to day 210 of development using a combination of single cell (sc) RNA-and ATAC-Seq, and ST approaches. ROs were categorized according to age in following developmental stages: early retinal development (day 10, 20, and 35), mid retinal development (day 45, 60, and 90), and late retinal development (day 150 and 210).

#### Early retinal development

**Figure 1:**
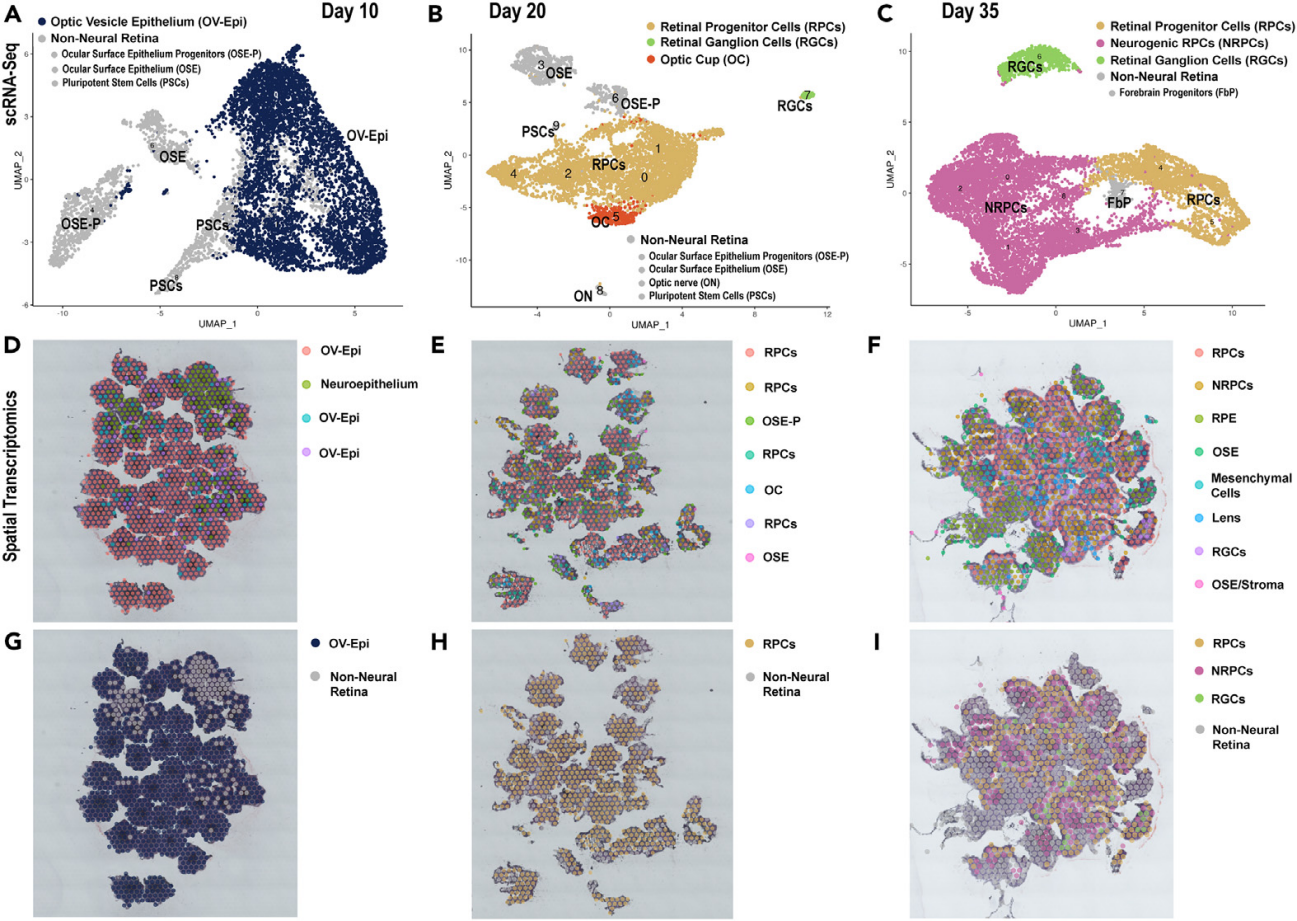
Early retinal organoid development (day 10–35) reveals key features of human retinogenesis, starting with optic vesicle formation, followed by the emergence of RPCs and RGCs (A‒C) UMAP plots of scRNA-seq in ROs at day 10 (A), day 20 (B), and day 35 of differentiation (C). (D‒I) Spatial localization of the individual (D‒F) and pooled (G‒I) clusters in ROs at day 10 (D and G), day 20 (E and H) and day 35 (F and I) of differentiation acquired from ST analyses. Cluster annotations and highly expressed markers are shown in Table S1 for scRNA-seq data and in Table S2 for ST data. For scRNA-seq 144 ROs were used at day 10/20 and 96 ROs at day 35. 40 ROs were used at day 10, 30 ROs at day 20 and 25 ROs at day 35 for ST. The scRNA-seq and ST were performed once at each time point. Related to Tables S1 and S2.

The early retinal development stage in ROs was commonly characterized by two key events: the eye field/optic vesicle formation and the emergence of RPCs/neurogenic RPCs (NRPCs) occurring in a sequential manner (Figure 1). The first time point, day 10 of differentiation, was dominated by optic vesicle epithelial clusters, revealing 73% and 87% abundance in scRNA and ST analyses, respectively (Figures 1A, 1D, 1G, and S1A). These clusters which covered nearly the whole of organoids (Figures 1G, 1D, and S1A) were characterized by high expression of several classical eye field transcription factors (TFs) such as *PAX6*, *NR2F1*, and *OTX2*^18^^,^^19^ (Tables S1 and S2) and typical optic vesicle markers including *FZD3*, *IGFBP5*, and *VIM*.^19^^,^^20^^,^^21^ Cell proliferation was a common feature of several optic vesicle clusters as demonstrated by high expression of *MKi67*, *TOP2A*, *TYMS*, *HELLS*, *MCM4*, and *HIST1H4C* (Table S1). As expected, a small cluster of undifferentiated PSCs was still present in day 10 ROs (Figure 1A; Table S1). Notably, two clusters of ocular surface epithelium (OSE), characterized by high expression of *KRT8*, *KRT18*, and *KRT19*, were identified by scRNA-seq, indicating that the culture conditions used for generation of ROs were also permissive for the emergence of other eye related cell types such as OSE cells (Figure 1A; Table S1).

Optic vesicle epithelium developed over time, and by day 20 of RO differentiation, only a small cluster (7.6% of total cells) of optic cup cells characterized by high expression of eye field and pigmentation markers (*MITF*, *VAX2*, *PMEL*, *LHX2*, and *PAX6)* was found alongside a large dominant heterogeneous pool of RPCs (Figures 1B, 1E, 1H, and S1B; Tables S1 and S2). RPCs were characterized by high expression of well-established markers including *ID3*, *ID1*, *SFRP2*, *VSX2*, *PAX6*, and *SIX3*^17^ and represented 74% of total cells (Figure S2A; Tables S1 and S2). At this stage of differentiation, RPC clusters revealed a broad distribution and were located all over ROs without any specific layering and/or spatial location (Figures 1E and 1H). A small but distinct RGC (RGC) cluster characterized by highly expressed genes such as *GAP43*, *SNCG*, *PRHP*, *STMN*, *ELAVL3*, *ELAVL4*, *NEFL*, *NEFM*, and *TBR1* was detected as early as day 20 of differentiation (Figure 1B; Table S1), which is slightly earlier than previously shown.^17^^,^^22^ This RGC cluster was only apparent in the scRNA-seq dataset due to either the current resolution of ST dots (50 μm diameter with a 100 μm center to center distance between spots) or the scarcity of this cluster. The RGC population increased over time, enabling the detection by both methods at day 35 of differentiation (Figures 1C, 1F, 1I, S1C, and S2B) and revealing their location at the basal side of ROs (Figures 1F and 1I). The most striking feature of this differentiation time point was the increased pool of neurogenic retinal progenitor cells (NRPCs), whose annotation was based on marker gene expression reported by Lu et al.^17^ (Figures 1C and S2B). Those give rise to all retinal cell types, except Muller glia and arise from the primary RPC population. The NRPCs were characterized by high expression of *VSX1*, *HES6*, *SOX4*, *SOX11*, *GADD45G*, *NEUROD1*, *NEUROD2*, *NEUROD6*, *GAP43*, *MEIS2* and displayed a distinct spatial profile to RPCs as revealed by ST analysis (Figures 1F, 1I, and S1C; Table S1). RPCs were found in an organized layer at the organoid’s apical edge, while NRPCs were predominantly located in the basal side (Figures 1F and 1I). Other non-retinal cell clusters including OSE progenitors, OSE, ocular surface stroma (OSS), mesenchymal cells, lens, and forebrain cells were also identified in day 20 and/or 35 (Figures 1, S1B, and S1C; Tables S1 and S2)*.*

In summary, single cell RNA-seq and ST demonstrate that the early development in ROs recapitulates the key events known from morphological and immunofluorescence studies of retinal development, starting with the eye field specification/optic vesicle formation, the emergence of RPCs and NRPCs, followed by the birth of an early born retinal cell type, the RGCs.

#### Mid retinal development

**Figure 2:**
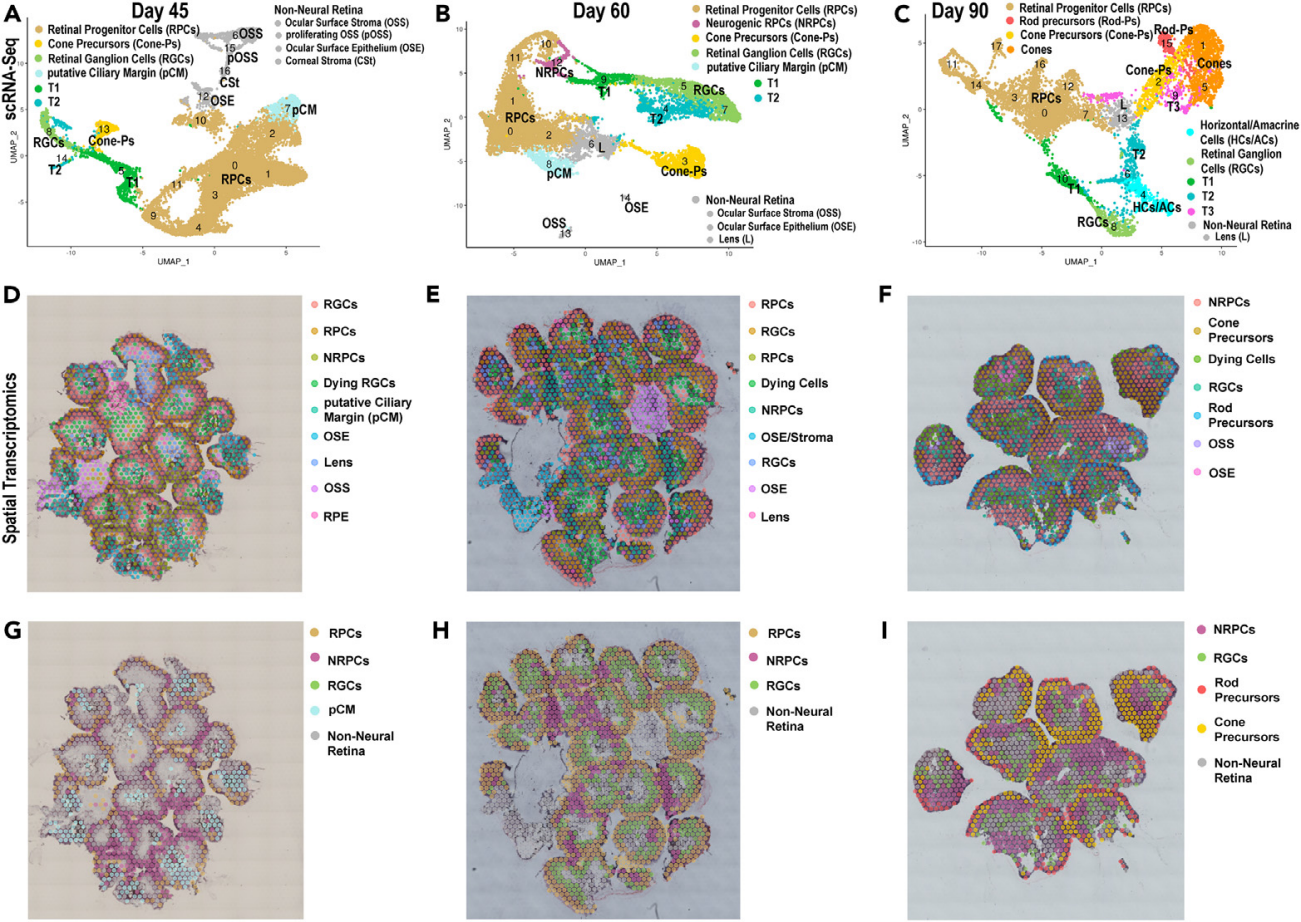
Mid organoid development (day 45–90) reveals the presence of pCM and specific localization of RPCs, NRPCS, RGCs and photoreceptor precursors (A‒C) scRNA-Seq UMAPs of hiPSC-derived ROs at day 45 (A), day 60 (B) and day 90 (C). (D‒I) Individual (D‒F) and pooled (G‒I) cluster location in ROs at day 45 (D and G), day 60 (E and H) and day 90 (F and I) of differentiation identified from the ST analyses. Cluster annotations and highly expressed markers are shown in Table S1 for scRNA-seq data and in Table S2 for ST data. For scRNA-seq 96 ROs were used for all days. 20 ROs were used at day 45/60 and 10 ROs at day 90 for ST. The scRNA-seq and ST were performed once at each time point. Related to Tables S1 and S2.

The mid developmental stage of ROs was characterized by the reduction of RPCs and NRPCs pool over time and changes in their spatial localization (Figures 2 and S2A). While RPCs were mainly located at the apical side of ROs at day 45 and 60 (Figures 2D, 2E, 2G, and 2H), NRPCs were found more basally toward the center of organoids at day 90 of differentiation (Figures 2F and 2I). Notably, we identified specific clusters (cluster 7 in the scRNA-seq and cluster 4 in the ST analyses at day 45, and cluster 8 scRNA-seq of day 60) (Figures 2A, 2B, 2D, S1D, and S2C) that were characterized by high expression of ciliary margin (*WNT2B*), eye field (*PAX6*), pigmented cell (*PMEL*, *TYRP1*) and RPCs (*ID3*, *ID1*, *SFRP2*, and *ZIC1*) markers^17^^,^^23^ (Tables S1 and S2). In a recent spatiotemporal phenotyping single cell analysis, we have observed the very same transcriptional signature in the ciliary margin zone of developing human retina and have shown that this region transiently harbors early RPCs.^24^ Using immunofluorescence and pulse labeling studies, Kuwahara and colleagues demonstrated the presence of a ciliary margin like stem cell niche in ROs, comprising a continuous neural retina epithelium with a small, pigmented domain of adjacent retinal pigment epithelium (RPE), which displayed the expression of both neural retina and RPE markers, with the capacity to expand the neural retina by *de novo* progenitor generation.^2^ Based on these findings we defined the clusters with high expression of ciliary, pigmented and RPCs markers, as pCM. In accordance, ST analysis revealed the location of this cluster at the very edge of day 45 ROs, mimicking the position of the ciliary margin zone in the developing human retina (Figure 2G).

The ST analyses of mid retinal development did not distinguish between the various types of NRPCs, however, the scRNA-seq revealed the emergence of three transient T1, T2, and T3 NRPCs, which have been shown to give rise to defined types of retinal neurons at specific developmental windows during human retinogenesis^22^ (Figures 2A–2C; Table S1). T1 cluster was located between the RPCs and retinal neurons and was defined as early as day 45 of RO differentiation (Figure 2A; Table S1). A T2 cluster was found next to T1 and RGCs at day 60 of differentiation, but at day 90 the very same cluster was found adjacent to a mixed population of horizontal/amacrine cells (Figures 2B and 2C). The T3 cluster became apparent at day 90 of RO differentiation and was located at the base of emanating cone and rod photoreceptor precursors clusters (Figure 2C; Table S1).

The RGCs clusters, the second largest besides RPCs/NRPCs in the mid-developmental stage reached its peak at day 60 and then decreased from this day onwards, reflecting the increasing cell type complexity of ROs (Figures 2B and S2B). Strikingly, our ST analyses provided evidence of dying RGCs at day 45 of RO differentiation (Figures 2D and S1D): these were characterized by the expression of many mitochondrial and ribosomal related genes in addition to RGCs marker genes such as *SNCG*, *NEFL*, *DCX*, and *STMN2* (Table S2). Those dying RGCs were located in the center of ROs (Figure 2D), reflecting the drastic reduction of RGCs during human retinal development. Dying cells located in the middle or organoids were also observed at day 60 and 90 of RO differentiation (Figures 2E and 2F), corroborating the presence of an apoptotic core often visible by bright field microscopy in ROs.

The first photoreceptor precursors to emerge were cone precursors: these were observed as early as day 45 of differentiation (cluster 13; Figure 2A) and expressed at high level cone-specific genes such as *THRB*, *CRX*, *PDE6H*, *PRDM1*, *DCT*, *IMPG2*, *OTX2* as well as some RGC-specific (*SNCG*, *DCX*, *STMN2*), horizontal cell specific (*ONECUT1*, *ONECUT2*, and *ONECUT3*), and NRPCs-specific (*ATHO7*, *NEUROD1*, *VXN*, *GADD45G*, *SCG*, *PCP4*) markers (Table S1). Similar cone cell clusters co-expressing RGC and interneuron markers were recently identified by our group in the retinas of 8–11 PCW of human specimens.^24^ This highlights the dynamic transcriptional profile of photoreceptor precursors during retinogenesis and points to the immature state of cones at this time of differentiation. The expression of NRPCs, RGCs and interneuron-related genes dissipated from cone precursor’s transcriptional profile at day 60 and 90 of RO differentiation, and cone precursor clusters were characterized by high expression of cone typical markers such as *PDE6H*, *ARR3*, and *THRB* (Tables S1 and S2). Cone precursors were observed spatially within ROs from day 90 of differentiation onwards, forming a thick organized layer at the apical side of ROs (Figures 2F and 2I). Rod precursors first identified at day 90 by both methods formed a thin layer above the cone cluster (Figures 2C, 2F, and 2I) and were characterized by highly expressed genes such as *NRL*, *RHO*, *ROM1*, *NR2E3*, *PDE6G*, *PDE6A*, *GNGT1*, *GNAT1*, and *GNB1* (Tables S1 and S2).

Apart from ROs containing cell clusters described previously, a different type of organoid was apparent from approximately day 30 of differentiation onwards, revealing a small retinal-like core with a big opaque cystic-like structure attached (Figure S3A), shown to contain OSS and OSE cell clusters by the ST analyses (Figures 2D, 2E, S1D, and S1E; Table S2). These organoids were named “eye-like organoids” hereafter and were further investigated using scRNA-seq and/or immunofluorescence (IF) analysis at day 45 and day 90 of differentiation, respectively (Figure S3; Table S3). Strikingly, all corneal cell types forming the three layers of the cornea were identified: the endothelium, the stroma, and the epithelium, which was further defined as conjunctival and corneal-limbal epithelium at day 45 (Figure S3B), and then further stratified to superficial corneal-limbal epithelium and basal corneal/conjunctival epithelium at day 90 (Figure S3C). The corneal endothelium cluster was characterized by high expression of *TWIST1*, *TWIST2*, *BMP4*, *PAX3*, *PDGFRA*, and *TAGLN* genes, while genes such as *LUM*, *COL1A1*, *COL1A2*, *COL3A1*, *COL5A1*, *COL6A2*, *OGN*, *POSTN*, *FOXC1*, *FBLN1* were highly expressed in the corneal stoma cluster (Table S3) while the conjunctival and corneal-limbal epithelial clusters were characterized by high expression of *KRT13* and *KRT15*, respectively (Table S3). Retinal cell clusters were also present including pCM, RPCs, RGCs, and cones as well as T1 at day 45 and a mixed cluster of horizontal and amacrine cells at day 90 (Figure S3B; Table S3).

IF analysis at the “eye-like organoids” at day 45 of differentiation confirmed the presence of conjunctival epithelium marked by the expression KRT13, limbal-corneal epithelium characterized by the expression of KRT15 at the apical edge of corneal organoids, and corneal stroma cells by the expression of lumican (LUM) inside the organoids (Figure S3D). At this time point many proliferative epithelial progenitor cells immunostained with MT2A and Ki67, and Np63, were detected within the eye-like organoids (Figure S3D). Alongside the corneal clusters, the organoids contained a small retinal core, showing thick Crx-positive rosette-like structures (Figure S3D; Table S3). The scRNA-seq also revealed the presence of lens clusters at day 90 in the “eye-like organoids” (Figure S3C), corroborated by the presence of CRYAA and CRYAB immunostained cells within the “eye-like organoids” (Figure S3D). The “eye-like organoids” didn’t develop further and were very rarely found after day 90, most likely due to an inappropriate differentiation media which was tailored for the neuronal retinal differentiation.

In summary, the mid-developmental stage was the most dynamical stage in where RPCs/NRPCs develop to transient cell progenitors, which then gave rise to retinal cell types such as interneurons and photoreceptor precursors. Notably, another type of organoid containing corneal, lens and retinal cell specific clusters were present at this stage, but their further survival was compromised by the inability of RO culture media to support the development of other eye cell types.

#### Late retinal development

**Figure 3:**
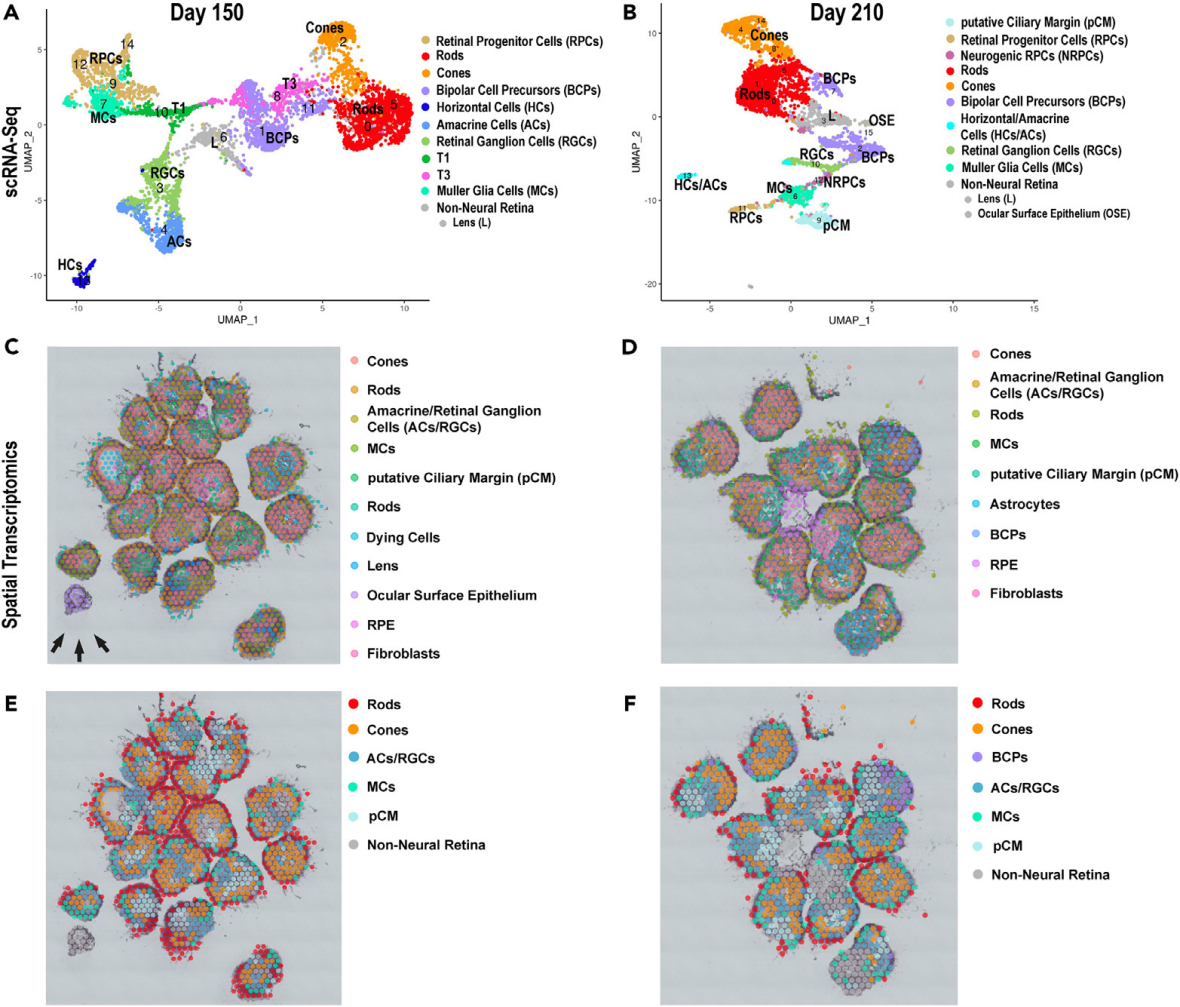
Late development of ROs (day 150–210) reveals maturation of photoreceptors, the appearance of BCs and MCs (A and B) UMAP plots of scRNA-seq of ROs at day 150 (A) and day 210 of differentiation (B). (C‒F) Localization of individual (C and D) and pooled (E and F) clusters in ROs at day 150 (C and E), day 210 (D and F) of differentiation based on ST analyses. Cluster annotations and highly expressed markers are shown in Table S1 for scRNA-seq data and in Table S2 for ST data. For scRNA-seq 96 ROs were used for all days. 15 ROs were used at day 150 and 210 for ST. The scRNA-seq and ST were performed once at each time point. Related to Tables S1 and S2.

The late developmental stages were characterized by the smallest pool of RPCs/NRPCs in comparison to other stages and an increasing population of mid to late born retinal cell types such as photoreceptors, bipolar (BCs), and Muller glia (MCs) cells (Figures 3, S1G, S1H, S2D‒S2F). ST demonstrated the presence of remaining RPCs within the pCM clusters (Figures 3E and 3F; Tables S1 and S2), which were characterized by high expression of ciliary muscle (*CPAMD8*, *COL9A2*, *ACTC1*, and *MYL1*)^25^ in addition to RPC markers (*ZIC2*, *SFRP2*, *IDE1*, and *ID3*) at day 150 and 210 of RO differentiation (Table S2). The T2 transient neurogenic cluster, which gives rise to horizontal and amacrine cells, was not present at this late stage of differentiation in accordance with the earlier emergence of these interneurons during the mid-stage of organoid development (Figures 3A and 3B). The T1 neurogenic progenitors which give rise to T3 and further differentiate into bipolar cell precursors (BCPs) and photoreceptors were identified by scRNA-seq at day 150 of differentiation but not at day 210 (Figures 3A and 3B), in accordance with the increased presence of photoreceptors and detection of BCPs during the late development stage (Figures 3, S2C, and S2D).

BCPs and MCs were first identified at day 150 of differentiation and were the last retinal cell type to emerge in ROs (Figure 3). This is consistent with birth dating and bulk RNA-seq studies, stating BCs and MCs to be of the latest cell types developing during retinogenesis.^26^^,^^27^ MCs were detected by both methods and were characterized by high expression of *RLBP1*, *GPX3*, *CRYM*, *CLU*, *DIO3*, *SLC1A3* (Tables S1 and S2). They were found in between the photoreceptor layer at the apical edge of organoids (Figures 3E and 3F), reflecting their apical processes, which form tight junctions with each other and/or inner segments of photoreceptors, establishing the outer limiting membrane. scRNA-seq data indicated the close relationship between MCs and RPCs clusters (Figures 3A and 3B) corroborating the MC's origin from RPCs shown earlier by Sridhar and colleagues.^22^ BCPs comprised 15% of total cells at day 150 and day 210 of differentiation (Figure S2D) and were detected by ST in the outer layer of ROs (Figures 3D and 3F).

Rods and cone photoreceptor clusters, first detected in the mid developmental stage, increased in occurrence (Figure S2C) and matured by developing inner/outer segments during the late development stage (Figures S4G and S4H). ST showed that rods were located close to the brush border of ROs and cones underneath those in the putative outer nuclear layer (Figures 3C–3F). Overall, ROs from day 150 and day 210 of differentiation revealed a comparable cellular composition, although some populations such as rod photoreceptors increased in occurrence at day 210 (Figure S2). Importantly, the scRNA-Seq demonstrated the presence of RGCs until the very last point of differentiation time point (day 210) (Figure 3B).

Similarly, to mid stages of differentiation, other cell types including fibroblasts, lens, astrocytes, and OSE cells were detected during the late differentiation (Figures 3, S1G, and S1H). ST demonstrated the presence of a few OSE organoids as a separate entity to ROs at day 150 of differentiation (black arrows in Figure 3C), however, these were not present any longer at day 210. Strikingly, astrocytes were detected in ROs of day 210 by ST (Figure 3D). In summary, ROs in the late developmental stage contained all the retinal cell types and only a small pool of RPCs. Moreover, this stage was characterized by an expansion of late born retinal neurons such as rods, BCPs and MCs as well as the overall maturation of retinal cell types (e.g., photoreceptors developed inner/outer segments, Figure S4).

### Pseudo-temporal analysis reveals a source of early RPCs in the putative ciliary margin and the transition of RPCs to neurogenic precursors and retinal neurons

**Figure 4:**
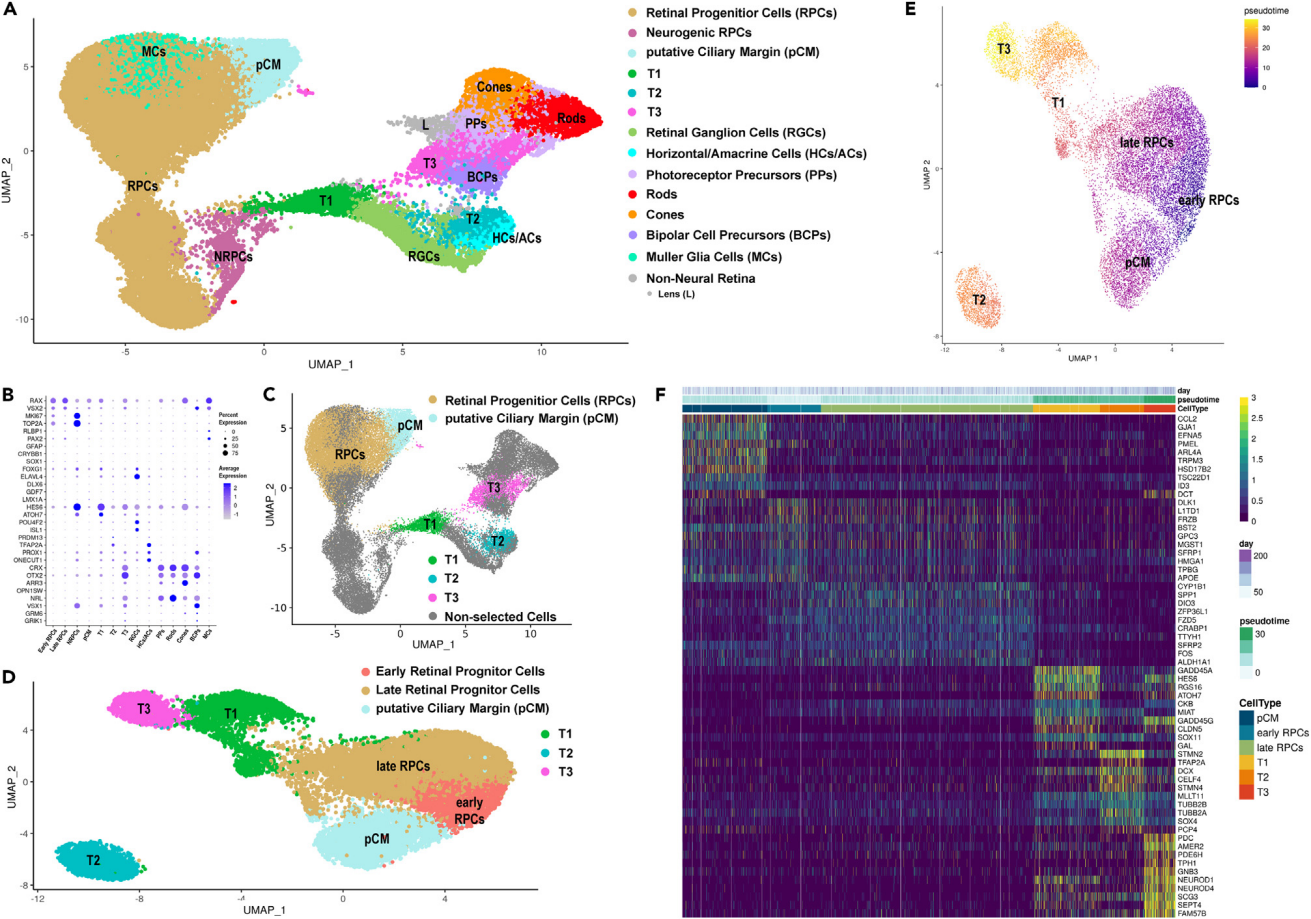
RPC identification and their developmental trajectories during RO development (A) UMAP plot of integrated scRNA-seq of ROs during development from day 10 to day 210 of RO differentiation. Cluster annotations and highly expressed marker genes are shown in Table S4. For ease of visualization, clusters of the same cell type are shown by the same color. (B) Dot plot presentation of highly expressed key marker for each retinal cell type in the integrated scRNA-seq data. (C) Individual selected clusters of integrated scRNA-seq retinal cells (A) used for pseudotime analyses. Highly expressed markers for each cluster along the pseudotime trajectory are shown in Table S4. (D and E) Pseudotime analysis demonstrating transition from pCM to early and late RPCs followed by T1 which further commits to either T2 or T3 transient neurogenic progenitors. (F) Gene expression heatmap, indicating similarities in gene expression patterns between the pCM and early RPCs, and between T1 and T3 transient neurogenic progenitors. Related to Table S4.

Transcriptomes of retinal progenitors and retinal neurons from all developmental stages (day 10–210) were integrated to better understand the molecular events that guide specification of RPCs and their differentiation (Figure 4A). After quality control, transcriptomes of 44,889 cells were obtained and merged, resulting in thirty-six transcriptionally distinct clusters visualized in UMAP. These clusters were defined using well-known and highly expressed marker genes for each retinal cell population (Table S4; Figure 4B). Of those, sixteen clusters were identified as RPCs, two as NRPCs, four clusters as neurogenic transitional populations (T1, T2, T3), two clusters as pCM, one cluster as lens and eleven clusters as retinal neurons such rods, cones, bipolar cells, horizontal/amacrine cells, RGCs, and MCs (Figure 4A; Table S4).

To investigate the temporal transition of RPCs to neurogenic transitional progenitors T1, T2, and T3, pseudo-temporal analysis of gene expression changes was performed (Figures 4C–4F). Eight RPCs and two NRPCs clusters characterized by high expression of genes such as *MKI67*, *TOP2A*, *UBE2C*, *CCND1*, *MCM3*, *UBE2C*, and *CENPF* were excluded from the analysis because of their mitotic/proliferating nature (Figure 4C; Table S4). As the pCM cluster possessed cells expressing early RPCs marker genes (Table S4), it was incorporated in the analysis (Figure 4C). The RPCs and the RPC-expressing cells in the pCM cluster shared common marker genes, typical of RPCs such as *ID1*, *ID3*, *SFRP2*, *ALDH1A1*, and *PCDH7*: in effect 28.3% of highly expressed genes in the pCM cluster were shared with early RPCs clusters. In addition, unique marker genes such as *ZIC1*, *FOXP1*, *PAX6*, and *HSPB* were highly expressed in the pCM (Table S4). In particular, high expression of *ZIC1* (zinc finger protein of cerebellum 1) was found mainly in the pCM cluster (Figure S5B). RNA-scope revealed the presence of *ZIC1* expressing cells exclusively at the very periphery of RO neuroepithelium (Figures S6A and S6B), lying adjacent to the *HES6* expressing cells that comprised the majority of retinal neuroepithelium, extending from the periphery toward the center of ROs (white arrowheads in Figure S6B).

ZIC1 is a member of the ZIC family of C2H2-type zinc finger proteins which play a fundamental role in various early developmental processes. Mutations in members of the ZIC family have been associated with a wide variety of congenital malformations, including Dandy-Walker malformation, holoprosencephaly, neural tube defects, and heterotaxy.^28^^,^^29^ Koso and colleagues^30^ showed that ZIC1 expression was restricted to SSEA1-positive RPCs in the peripheral region (ciliary margin) of the mouse retina, which has been postulated to contain stem cell like populations that can further develop to RGCs.^31^ A similar stem cell niche with the potential to expand the neural retina was found in a ciliary margin like zone of ROs;^2^ hence, we hypothesize that the pCM cells that express RPCs markers may contribute to retinal neurogenesis, giving rise to early RPCs of the neural retina. This was corroborated by our pseudotime analyses, indicating the start of the developmental trajectory in pCM, leading to early RPCs (characterized by high expression of *ID3*, *MGST1*, *SFRP1*, *APOE1),* followed by late RPCs (characterized by high expression of *SPP1*, *DIO39*, *FOS*, *CRABP1*, and *FZD5)* and the T1 transient neurogenic precursors (Figures 4D–4F; Table S4).

**Figure 5:**
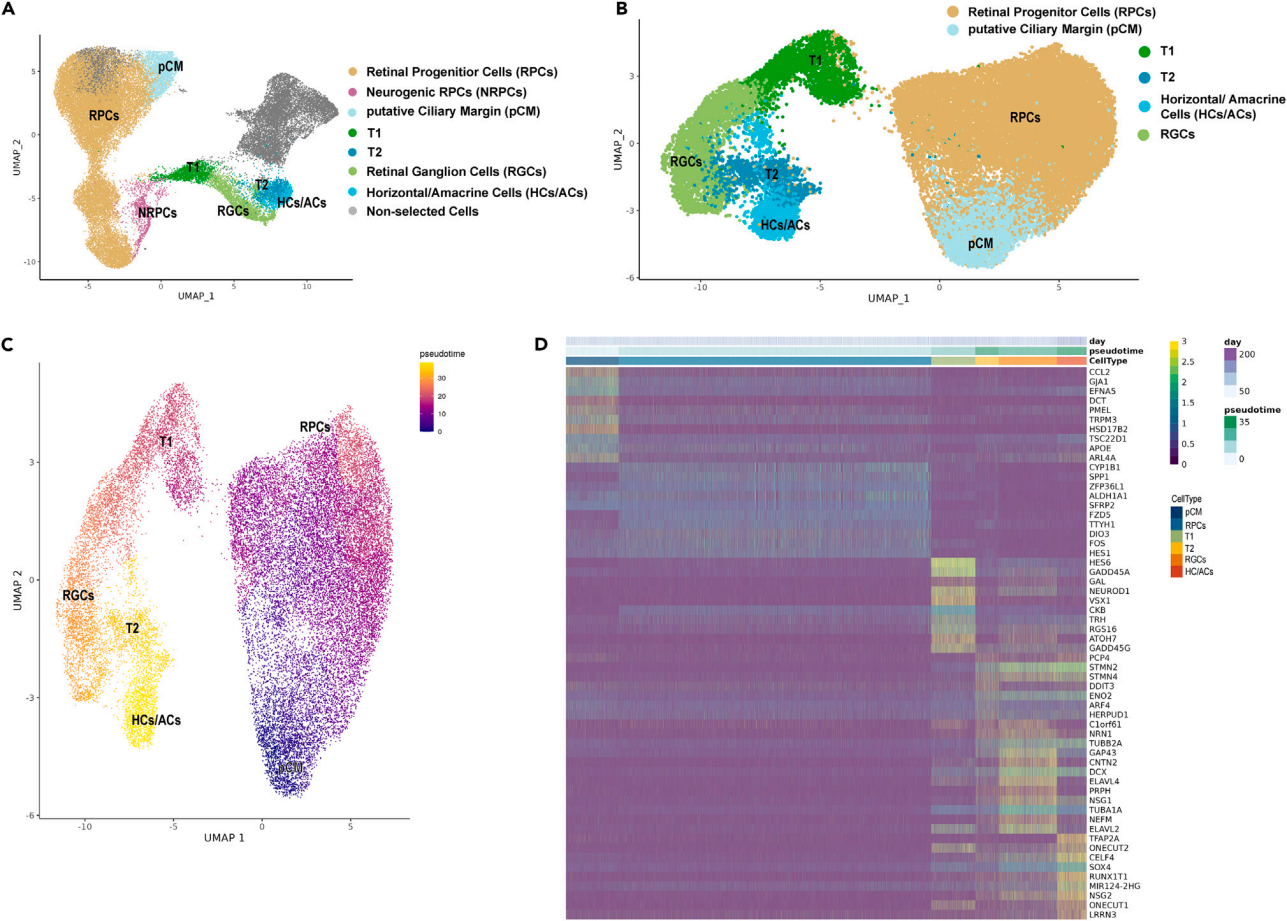
Pseudotime trajectory analysis indicates the emergence of horizontal and amacrine cells via T1 and T2 neurogenic progenitors (A) pCM, RPCs, T1, and T2 transient neurogenic cells and horizontal/amacrine cell clusters were selected from scRNA-seq and used for pseudotime analyses. Highly expressed genes for each cluster are shown in Table S4. (B and C) Pseudotime analysis revealing transition from pCM to RPCs, T1 and T2, further giving rise to horizontal/amacrine cells. (D) Gene expression heatmap, showing some overlapping gene expression between T1 and T2 transient neurogenic progenitors as well as between T2 and RGCs, and horizontal/amacrine cell clusters. Related to Table S4.

The T1 cluster led to two further transitional populations (T2 and T3, Figure 4D); in accordance, 13.6% of T1’s highly expressed markers were shared with T2 and T3 clusters (Figure S5C; Table S4). RGCs characterized by high expression of *GAP43*, *PRPH*, and *SNCG*, emanated from the transitional cluster T1 (Figure 5; Table S4), confirming recent transcriptomic and birth dating studies of the human retina and ROs.^22^^,^^32^^,^^33^ T2 gave rise to a mixed cluster of horizonal and amacrine cells characterized by high expression of *TFP2A*, *ONECUT1*, *ONECUT2*, *MEIS2*, *PAX6*, and *GRIA4* (Figure 5; Table S4).

**Figure 6:**
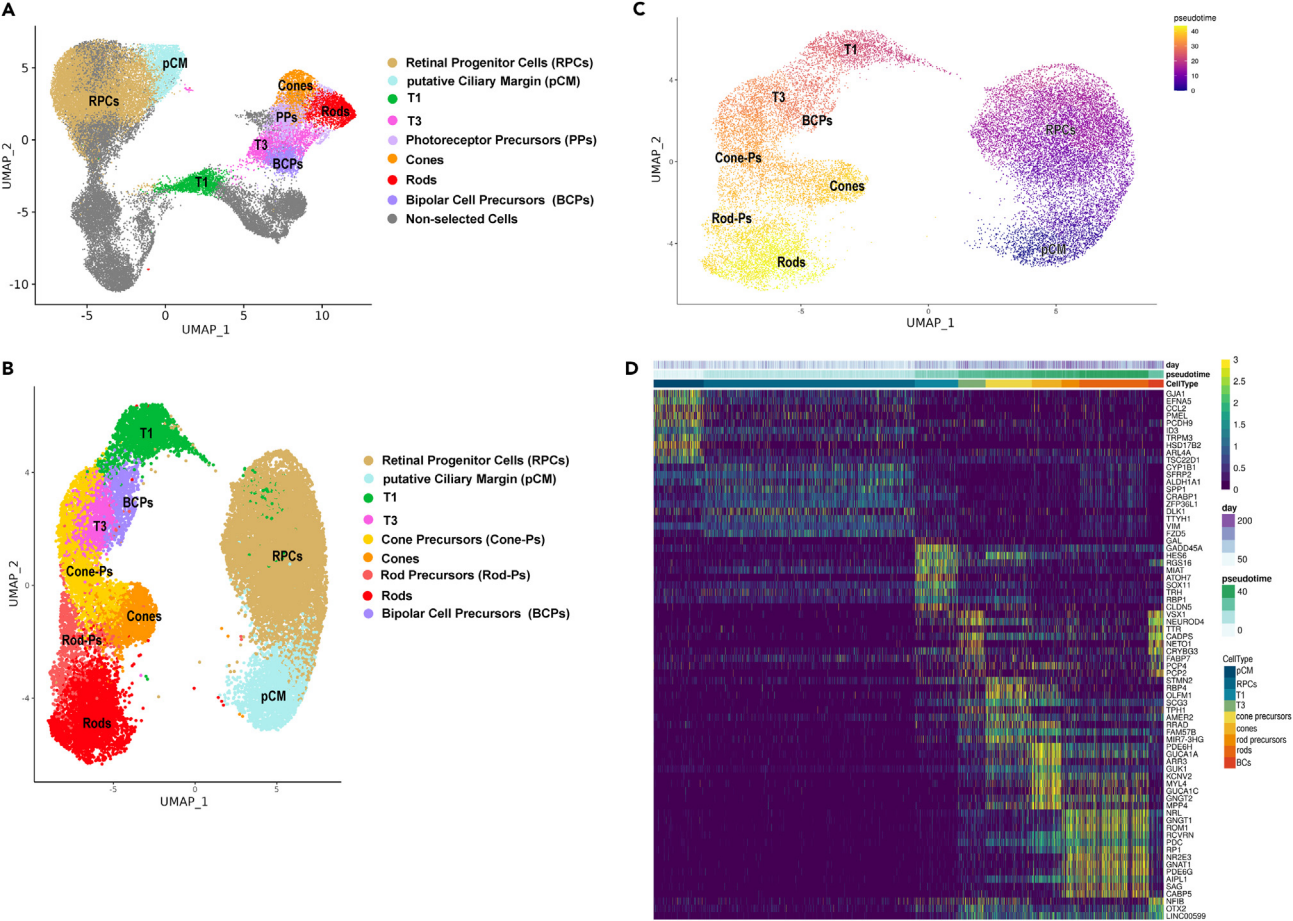
Emergence of photoreceptor and bipolar cells via T1 and T3 neurogenic progenitors (A) UMAP plot showing selected cluster of integrated scRNA-seq (Table S4) used for pseudotime analyses. Highly expressed marker genes for each cluster along the pseudotime trajectory are shown in Table S4. (B and C) Pseudotime analysis revealing transition from pCM to RPCs, T1 and T3, further giving rise to cone and rod photoreceptors and bipolar cells. (D) Gene expression heatmap revealing distinct expression patterns of T1 and T3 progenitors and photoreceptors, while BCPs display transcriptional similarity with transient neurogenic cells, T1 and T3, suggesting the lack of mature bipolar cells in ROs. Related to Table S4.

Photoreceptor precursors and BCs originated from the transitional population T3 (Figure 6), confirming previous studies.^22^ Although the BC cluster expressed several key marker genes such as *VSX1*, *NEUROD4*, *CADPS*, *PDRM8*, *VSX2*, and *OTX2*, it also revealed strong transcriptional similarity to T3 (Figure S5D). Based on this evidence, we concluded that ROs in this study contained BCPs, which explains their early temporal emergence (next to T3) in the pseudotime analyses (Figures 6B and 6C). Cone precursors expressing *DCT*, *THRB*, *PDE6H*, *HES6*, *SCG3*, *NEUROD4*, NEUROD1, *VXN*, and *CRX* genes led to mature cones, characterized by high expression of *PDE6H*, *ARR3*, *GUCA1A*, *GNGT2*, *GUK1*, *TULP1*, and *AIPL1* (Figure 6D; Table S4). Rod precursors expressing *NRL*, *RECOV*, *NR2E3*, *AIPL*, *NEUROD1*, *ROM1*, and *CRX*, developed slightly later than cone precursors and matured over time to rods (Figures 6B–6D), which were characterized by high expression of genes such as *PDE6G*, *ROM1*, *GNGT1*, *GNAT1*, *NR2E3*, *RHO*, and *CNGB1* (Table S4).

In conclusion, RO development recapitulated closely human retinogenesis, starting with RPCs which differentiate into the transitional populations T1, T2, and T3, followed by the appearance of all retinal neurons in a sequentially and precise order. The first-born retinal cell type were RGCs followed by horizontal/amacrine cells, cone precursors/cones, rod precursors/rods, BCPs, and MCs. Remarkably, we demonstrate that the pCM region of ROs may have the potential to give rise to early RPCs, indicating their putative contribution to human retinogenesis.

### Chromatin accessibility of retinal organoids is highly correlated to the developing human retina

Single cell ATAC-s,eq of ROs at day 10, 20, 35, 45, 60, 90, 150, and 210 was carried out to assess chromatin accessibility during RO differentiation (Table S5). In total, 84,833 cells were captured using the 10XGenomics Chromium Single Cell ATAC Library and Gel Bead Kit (version 1.1). We used CellRanger to align reads and carry out data processing, and Signac/Seurat and Monocle for quality control, differential accessibility, motif enrichment, and trajectory analyses. The scATAC-seq clusters for each stage of ROs differentiation were identified using the corresponding scRNA-seq datasets (Figures S7 and S8). Similarly, to scRNA-seq analysis, defined clusters of OSE, corneal stroma, fibroblasts, and corneal endothelium were easily identified: those were present mostly in the early stages of RO differentiation (up to day 60, Figures S7A‒S7E) and were much less evident at the later stages of differentiation, probably due to the incompatibility of our retinal culture media to support corneal cell survival and differentiation. Cell clusters representing optic vesicle, RPCs and NRCPs were found in the very early stages of RO differentiation (day 10 and 20) and the earliest born retinal cell types, RGCs were first identified at day 10 of RO differentiation (Figures S7A and S7B). Notably, cell clusters of pCM, harboring signatures of ciliary body, RPCs and pigmented cell markers were identified from day 45 of RO differentiation (Figure S7D; Table S5), corroborating both the scRNA-seq and the ST analyses. Notably, the three clusters of transient neurogenic progenitor cells, namely, T1, T2, and T3 were present from day 45 of RO differentiation (Figures S7D and S7E). All the other retinal cell types emerged in an orderly fashion with horizontal and amacrine cell clusters appearing at day 35 of RO differentiation, cones from day 60, and rods, BCs and MCs from day 90 of differentiation (Figures S7C‒S7F and S8).

Following filtering and quality control, 37,522 retinal cells from day 10–210 ROs with a 12,179 average median fragments per cell were integrated (Figure S8C). We used “bedtools merge” to create a shared peak bed file, “cellranger reanalyse” to create a shared peak set with a total of 314,490 chromatin accessibility peaks, and Signac to create a gene activity matrix. Cell clustering based on chromatin accessibility peaks resulted in 17 clusters, which were identified by assaying for increases in DNA accessibility near previously known marker genes for each specific cell type (Figure S8C; Table S5). Those included the pCM and early and late RPC clusters, as well as the T1 and T3 transient neurogenic progenitors, MCs and all retinal neurons (Figure S8C). We did not identify a T2 cluster, which could be due to the transient nature of these progenitors and/or the relatively low cell number obtained for data integration following filtering and quality control.

**Figure 7:**
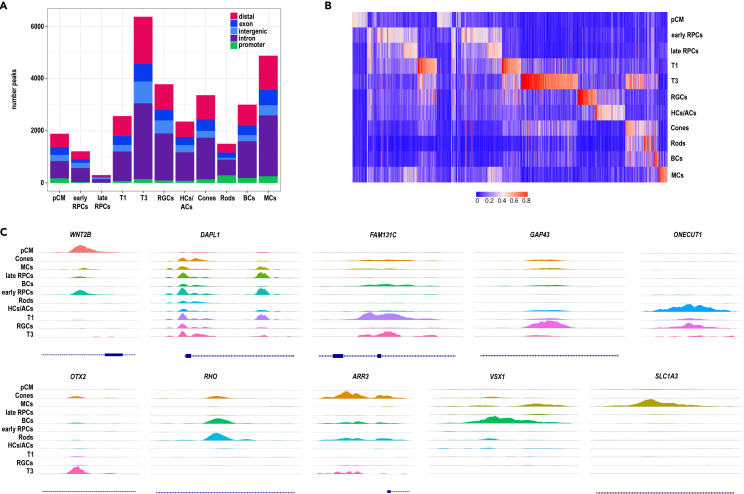
Single cell ATAC-seq analysis of ROs indicates cell type specific chromatin accessibility profiles (A) The number and type of chromatin accessibility profiles for each retinal cell type found in ROs. Data are based on integrated scATAC-seq shown in Table S5. (B) Differentially accessible of chromatin accessibility peaks for each cell type. (C) Representative examples of chromatin accessibility peaks for each retinal cell type individually. Each track represents the aggregate scATAC signal of all cells from the given cell type normalized by the total number of reads in TSS regions. For scATAC-seq-seq 96 ROs were used for all days. The scATAC-seq was performed once at each time point. Related to Tables S6.

The DNA accessibility peaks were classified using annotation from cellranger as follows: associated with promoters (if found within −1000 to +100 bp of the transcription start sites), exons, introns, distal (if found within 200 kb of the closest transcription start site), or intergenic regions (if not mapped to any genes) (Figure 7A; Table S6). This analysis enabled the identification of cell type specific regions of accessibility for all retinal neurons arising in the ROs as well as RPCs and transient neurogenic progenitors T1 and T3 (Figure 7B). We observed scATAC-seq marker peak enrichment of cell type specific markers as follows: *WNT2B* in the pCM, *DAPL1* in the early RPCs, *FAM131C* in T1, *GAP43* in RGCs, *ONECUT1* in horizontal and amacrine cells, *OTX2* in T3, *RHO* in rods, *ARR3* in cones, *VSX1* in bipolar cells and *SLC1A3* in Muller glia cells (Figure 7C).

**Figure 8:**
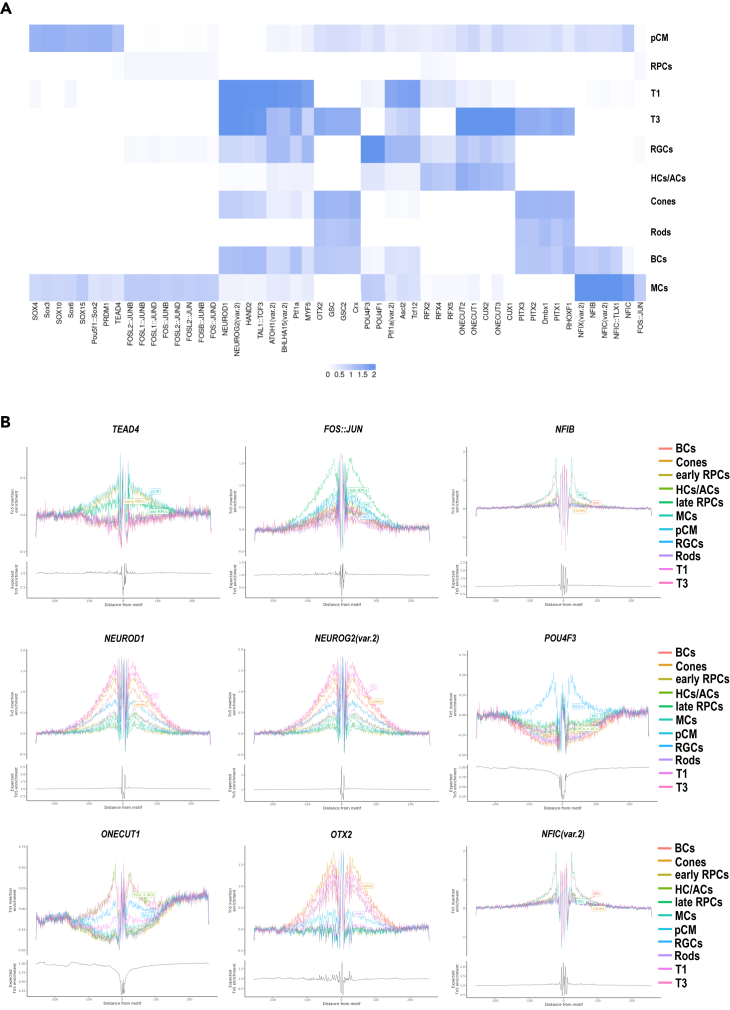
Motif analysis of accessible DNA peaks predicts cell-type specific transcription factors in the retinal organoid development (A) Heatmap of transcription factor binding motifs enriched in each cell type. Darker colors represent more significant enrichment. (B) Footprinting analysis of selected transcription factors predicted to display a significant enrichment in retinal cell types. Related to Tables S6.

We identified TF binding motifs using Signac, which were further validated by footprinting analysis (Figure 8; Table S7). In the pCM, we identified binding motifs for members of the SOX, PRDM, and TEAD family of TFs (Figures 8A and 8B), corroborating the binding motifs identified by our group in RPCs of fetal retina.^24^ Shared TF binding motifs were observed between RPCs and MCs for FOS/FOSL:JUN TFs, most likely reflecting the similarity in transcriptional profile between these two cell types as reported recently.^17^ In accordance with generation of T3 progenitors from T1, we identified a set of shared TF binding motifs for NEUROD1, NEUROG2, HAND2, and TAL1:TCF3 (Figures 8A and 8B). The RGCs displayed strong POU4F family member TF binding motifs, while horizontal and amacrine cell clusters were defined by ONECUT1, RFX, and CUX TF family members binding motifs (Figures 8A and 8B), corroborating our recent data in the developing human fetal retina.^24^ As highlighted by our scRNA-seq, cones, rods, and bipolar cells are derived from the T3 neurogenic progenitors; it is therefore not surprising to observe shared binding motifs for TFs such OTX2, GSC2, PITX3, PITX2, CRX, and DMBX1 (Figures 8A and 8B), which are well described in the literature for their expression and/or role in photoreceptor specification.^34^^,^^35^^,^^36^^,^^37^^,^^38^ Enrichment of NFI and NFX family members binding motifs was observed for MCs and BCs (Figures S8A and S8B), consistent with their suggested role in the specification of the late-born cell types in the retina.^17^ These findings were largely corroborated by the SCENIC analysis, which in addition to revealing the specific TF binding sites in key retinal cell types, also demonstrated an overlap in TFs binding motifs for the pCM, early and late RPCs (Figure S9A).

To identify gene regulatory networks (GRNs) that govern RPCs diversification and their differentiation, we used the Qiagen Ingenuity Pathway Analysis (IPA) upstream analysis tool combined with overlay analysis of DA peaks as well as SCENIC. In early RPCs, we predicted the activation of proliferation related upstream regulators such as MYC and MYCN, which activate expression of early RPCs (e.g., *HMGA1*) and inhibit expression of late RPCs genes (e.g., *Cyclin D1*) (Figure S10A; Table S8). In late RPCs, several upstream regulators were identified including FGF2, TGFβ, IGF-1, and YAP1, regulating the expression of a common pool of target genes that specify late RPCs (e.g., *SOX9*, *VIM*, *CCND1*; Figure S10B). When early and late RPCs were pooled together, three key regulators, namely FOS, FOSB, and HMGA2 were identified (Figure S9B). Among the predicted inhibited upstream regulators, we found key transcription factors such as THRB and RB1 (Table S8),^39^^,^^40^ known for their role in the development of cone photoreceptors and retinal neurogenesis, respectively (Figure S10C). As RPCs differentiated to retinal neurons, a key regulator ATOH7 was identified, corroborating the scRNA-seq data specifying high expression of this gene in the T1 transient neurogenic progenitors (Figure S9C). During RPC differentiation we observed that the majority of predicted upstream regulators harbored an inhibitory role, perhaps demonstrating a restriction in gene expression profiles, which coupled with activation of lineage restricted TFs direct the differentiation to photoreceptors, RGCs and retinal interneurons. For example, CRX and NEUROD1 were predicted as upstream regulators in photoreceptors (Table S8), activating expression of key genes involved in phototransduction such as *PDE6B*, *PDE6A*, *RHO*, and *SAG* (Figures S9D and S10D). The major share of predicted inhibited upstream regulators was taken by miRNAs, which suppress activation of genes necessary for retinal cell fate specification. Exemplary among those were miR-6825-5p in cones (Figure S10E) and miR-153-3p in bipolar cells (Figure S10F), regulating the expression of genes encoding key proteins for cone photoreceptor genesis and function (e.g., *RAX2*, *RXRG*) and bipolar cells (e.g., *NEUROD4*, *NFIB*), respectively. In addition, the SCENIC analyses revealed the close connection between the GRNs that govern T1 and photoreceptors (Figure S9E), corroborating the pseudotime analysis showing the differentiation of photoreceptors via the T1-T3 lineage (Figures 6B–6D).

The analysis of TF binding profiles and upstream regulators showed multiple similarities between the developing human fetal retina samples analyzed by our group recently^24^ and ROs studied herein. To better assess the similarity in chromatin accessibility landscape between the developing retina and ROs, we extracted the metadata from each dataset, created a unified set of overlapping differential accessibility peaks which were integrated using Harmony to remove batch effects (Figure S11A; Table S9). Next, we ordered the human fetal retina and ROs samples in pseudotime using Monocle, revealing a nice progression from day 10 to day 210 of development with ROs being most closely situated to the equivalent age fetal retina samples (Figure S11B). This was further exemplified by Spearman analysis which demonstrated 0.89 correlation between the day and pseudotime of the sample (data not shown). Pseudotime analysis for each cell type demonstrated a slightly earlier emergence of T1 neurogenic progenitors in the human developing retina samples, but similar dynamics for the T2 and T3 progenitors which emerge from T1 (Figure S11C). In contrast the RGCs and cones emerged earlier in the ROs, rods and BCs later, while horizontal and amacrine cell emergence followed the timeline of human fetal retina development (Figure S11C). To compare the chromatin accessibility for each cell type, we cross-correlated the pseudo-bulk DA sample in the RO to the equivalent dataset obtained from our single cell ATAC-seq analysis of developing human retina.^24^ All cell types displayed a strong correlation between ROs and human fetal retina with a Spearman coefficient ranging from 0.66 to 0.69 in amacrine cells and cones, respectively, to 0.7 in BCs, 0.76 in rods, 0.77 in horizontal cells, 0.79 in MCs and 0.89 in RGCs (data not shown). Together these data demonstrate that the chromatin accessibility of ROs is highly correlated to the developing human retina, but with some differences in the temporal emergence and abundance of the retinal progenitors and mature cell types.

## Discussion

ROs generated from human PSCs provide a tractable platform for studying early retinal development and disease. Several recent studies have used scRNA and/or ATAC-seq to reveal cell type composition in ROs and to identify cell specific *cis*-regulatory elements and GRNs.^10^^,^^12^^,^^14^^,^^15^^,^^22^^,^^41^^,^^42^^,^^43^ Recently Wahle and colleagues (2023) developed a multiplex immunohistochemistry approach to establish a spatial retinal organoid map of retinal cells during development, but this was based only on expression of 63 antibodies covering the majority of the retinal cell types.^44^ Herein, using a combination of scRNA-seq and ST approaches, we present for the first time a genome wide, single cell spatiotemporal transcriptome map of RO development, revealing the sequential and precise order of all retinal neuron emergence and their specific localization, which closely recapitulates human retinogenesis. Importantly, we provide evidence for the existence of a pCM domain at the very edge of ROs, from where a population of early RPCs is likely to arise. Combining the scRNA-with scATAC-seq data, we were able to reveal cell type specific TF binding motifs on accessible chromatin at each stage of RO development and to show that chromatin accessibility of ROs is highly correlated to the developing human retina, but with some differences in the temporal emergence and abundance of some of the retinal cell types.

The human retinal ontogenesis starts very early during human development. At 2 weeks post conception the optic vesicle evaginates to form the optic cup, whose inner layer differentiates into neural retina.^45^ These processes represent fundamental events for retinal ontogenesis, however, due to scarcity of available human retinas of this age, most of our molecular and developmental data so far have been acquired from animal models. To fill this gap in our understanding of retinal development, we have used very early ROs (day 10 and 20), revealing for the first time the molecular signature of optic vesicle and optic cup neuroepithelium at the single cell level, and most importantly their spatial localization, thus providing novel information and early molecular markers for assessing retinal development *in vitro* and *in vivo*. We were able to detect a large pool of RPCs at day 20 of RO differentiation using both scRNA- and ATAC-seq. ST revealed that the RPCs were initially located all over the ROs, but from day 35 the RPCs localized at the apical edge of the organoids, while the NRPCs were predominantly found on the basal side of the organoids. Notably from day 45, we were able to identify with all three methods a domain located at the very edge of ROs with transcriptional signatures of RPCs, ciliary body and pigmented epithelium, which we defined as a pCM based on similar transcriptional signature we revealed by spatiotemporal single cell analyses of developing human retina in a recent study.^24^ A similar entity was described by Kuwahara and colleagues (2015) who used IF imaging and pulse labeling studies to show that the pCM region can expand the neural retina by *de novo* progenitor generation.^2^ In accordance with this, our pseudotime analysis positions the pCM at the start of RPCs pseudotime indicating that some of the earliest RPCs in the organoids are likely to emerge from this region. If this were to be the case, the question remains of why this domain was not detected at the earlier stages of organoid development? Could this be due to two separate sources of RPCs: one directly arising from differentiation of optic cup to retinal neuroepithelium and the second from the pCM domain which is established slightly later in development? Or could this be due to the small size of pCM in earlier organoids? To answer this question, lineage tracing combined with single cell RNA-seq and ST need to be performed as recently demonstrated for first heart field predominance of human PSC differentiation.^46^

RGCs developed in ROs have been reported to be progressively lost in long-term cultures,^5^ probably due to demand for additional reagents in the culture media that promote their survival. In contrast we were able to detect RGC clusters from day 10 and day 20 until the end of the differentiation time point (day 210) by both scATAC- and scRNA-seq methods, respectively, and able to locate them predominantly on the basal side of ROs using ST. This could be due to the presence of IGF-1 in our culture media, which has been shown to enhance RGC survival *in vivo.*^47^ Strikingly, we were able to detect dying RGCs in the center of ROs, likely reflecting the drastic reduction of RGCs during human retinal development. The similarity of RGCs to their *in vivo* counterparts in the developing human retina was further demonstrated by a very strong correlation (0.89) revealed by the comparison of the accessible chromatin peaks.

Horizontal and amacrine cell clusters were identified by both scRNA- and ATAC-seq methods from day 60 and day 35 of RO differentiation, respectively, but not always as separate clusters, which could be due to the high similarity in their transcriptional and chromatin accessibility profiles and/or low abundance in organoids. This was also the case for the transient neurogenic progenitor population T2, which give rise to both of these cell types, but was often difficult to be identified as a separate cluster in the later stages of RO differentiation (day 150 and day 210). Comparison of abundance of these cell types using the chromatin accessibility and transcriptional data demonstrated a much lower percentage of horizontal, amacrine and T2 progenitors in the ROs compared to human fetal retina (data not shown), suggesting that the 3D environment and/or culture conditions are not fully supportive of the development of these progenitors/interneurons. These findings corroborate published evidence showing a disorganization of the inner layer of organoids at approximately day 70 to day 90 of development.^22^ We also could not detect horizontal or T2 cell clusters in our traditional ST analyses, which could be due to the ST data not being single cell resolution, but a group of cells. To this end, we performed deconvolution of the cell type proportions that are present in day 90, 150, and 210 of RO differentiation (Figure S12) and we were able to detect not only horizontal and amacrine cells, but also the transient neurogenic progenitors, T1, T2, and T3.

Our data demonstrate that cones arise slightly earlier and rods slightly later in ROs, compared to their emergence *in vivo*. Both types of photoreceptors were localized by ST in the apical layer of ROs, with rods located close to the brush border of ROs and cones just underneath those. BCs and MCs were the last cell types to develop in ROs, corroborating previously published data for ROs and fetal retina.^10^^,^^17^^,^^33^ BCs developed slightly later in ROs compared to human fetal retina and demonstrated a high transcriptional similarity with T3 progenitors, which led us to suggest that these more likely represented BCPs, rather than mature BCs.

Recently Fernando and colleagues (2022) described the formation of brain and ROs from PSCs connected by nerve-like axonal projections of optic origin using a 2D-3D method.^48^ While such organoids were not detected in our culture system, another type of organoid which contained a retina-like core, but also an opaque cystic-like structure within which corneal epithelium, stroma, endothelium, and lens cell clusters was detected, corroborating previously published data by our group.^49^ Given the presence of corneal, retinal, and lens cells within the same organoid, we refer to those as “eye-like organoids.” These organoids did not survive beyond day 90 of differentiation, suggesting that an adapted media composition is necessary to further promote their survival and maturation.

The presence of astrocytes in the retina was first described by Cajal,^50^ however, their origin has remained under discussion for a long time, with some proposing the astrocytes to be derived from transformed MCs and others suggesting that these cells may arise *in situ* from the retinal neuroepithelium^51^ as they do in spinal cord grafts in avians.^52^ Strikingly, ST revealed the presence of a small astrocyte cluster in day 210 ROs, characterized by high expression of GFAP and VIM, which may suggest that these could represent immature astrocytes.^53^ In rodents, astrocytic cells migrate into the retina through the optic nerve head and then spread toward the periphery across the nerve fiber layer.^53^ Moreover, the optic nerve serves as the source of astrocyte progenitors, thus the presence of the optic stalk is critical for astrocyte development. We did not detect cell clusters with transcriptional signatures of optic stalk at day 210 of differentiation; thus, we believe that origin of astrocytes in humans may differ from rodents, however, this remains to be further investigated. Our findings echo recent published data showing the presence of donor derived astrocytes into retinas of mice transplanted with micro-dissected multi-layered retinal fragments from human ROs.^54^ It not clear if astrocyte progenitors were present in the ROs in small numbers or transplantation induced *trans*-differentiation of neural retinal cells into astrocytes. However, given the presence of astrocytes in ROs studied herein, we are tempted to speculate that astrocyte progenitors do arise in ROs, although their cell of origin remains as yet unknown.

Application of all three techniques on ROs samples of the same age enabled us to compare and contrast our results. Similarly, to our findings in human fetal retina^24^ we observed that scATAC-seq preceded cell date identification compared to scRNA-seq. For example, a small RGCs cluster was identified at day 10 ROs in the scATAC-seq, but only in day 20 by scRNA-seq. Likewise, BCs and MCs were found at day 90 in scATAC-seq while scRNA-seq indicated their emergence at day 150. This suggests that the chromatin accessibility changes, and TF binding occur in advance of cell specific gene expression changes that drive retinogenesis. Notably, some cell types (e.g., lens) which are resistant to single cell dissociation with enzymes tailored for retinal neurons, were detected by ST, but not by scRNA-seq analyses. We thus conclude that combination of several single cell analyses is necessary to fully map the composition of all cell types within the ROs as well as their spatial localization.

### Limitations of the study

Due to relatively high cost of single cell analyses, all the results presented in this study are derived from ROs generated from a single human iPSC (hiPSC) line, with validations performed in ROs generated from one human embryonic stem cell (hESC) line. It would be useful to expand all the three single cell analyses on ROs generated from additional PSCs grown under the same as well as different culture conditions. We also used the Visium ST system which has 4992 barcoded gene expression spots per capture area where each spot has diameter of 55μm with a 100μm center to center distance between spots. This was insufficient in our experience to define all the progenitor and retinal cell types. It is thus desirable to use a higher definition ST method and combine it with protein detection to better define rarer cell types and localize them within the ROs. Cell barcoding combined with single cell analyses are currently being tested in a few model systems^46^^,^^55^: these methods combined would provide better insights into sources of RPCs and their differentiation trajectory. Despite these minor limitations, our work provides the scientific community with a powerful spatiotemporal single cell atlas of human PSCs undergoing differentiation to laminated ROs.

## STAR★Methods

### Key resources table

**Table:** 

REAGENT or RESOURCE	SOURCE	IDENTIFIER
**Antibodies**		

CRYAA	gift from Roy Quinlan	
CRYAB	Abcam	Cat# ab76467; RRID: AB_1523120
CRX	Abnova	H00001406-MO2; RRID: AB_606098
Ki67	Abcam	Cat# ab15580; RRID: AB_443209
KRT13	Abcam	Cat# ab92551; RRID: AB_2134681
KRT15	Abcam	Cat# ab52816; RRID: AB_869863
Lumican	Santa Cruz Biotechnology	Cat# sc-166871; RRID: AB_10607817
MT2A	Sigma-Aldrich	Cat# SAB1402848; RRID: AB_10737934
NP63	Abcam	Cat# ab735; RRID: AB_305870
Goat Anti-Mouse Alexa488	Jackson Immuno Research Laboratories	Cat# 115-545-146; RRID: AB_2307324
Goat Anti-Rabbit Cy3	Jackson Immuno Research Laboratories	Cat# 111-165-003; RRID: AB_2338000

**Chemicals, peptides, and recombinant proteins**		

Accutase	Thermo Fisher Scientific	Cat# A1110501
B27 Supplement	Thermo Fisher Scientific	Cat# 17504001
Bluing Buffer	Dako	Cat# CS702
Bovine serum albumin (BSA)	Sigma-Aldrich	Cat# A2153
Chemically Defined Lipid Concentrate	Thermo Fisher Scientific	Cat# 11905031
DMEM/F-12	Thermo Fisher Scientific	Cat# 11320033
Ethanol	VWR	Cat# 85651.320
Eosin solution	Sigma-Aldrich	Cat# HT110216
Fetal Bovine Serum (FBS) used in combination with the Neurosphere dissociation kit	Miltenyi Biotec	Cat# 130-091-376
Fetal Bovine Serum (FCS) used for RO differentiation	Thermo Fisher Scientific	Cat# A5256801
Fungizone	Thermo Fisher Scientific	Cat# 15290018
GlutaMAX™ Supplement	Thermo Fisher Scientific	Cat# 35050087
Hanks' Balanced Salt Solution	Thermo Fisher Scientific	Cat# J67763-AP
Hematoxylin	Dako	Cat# S3309
Hoechst	Thermo Fisher Scientific	Cat# H3570
IGF-1	Merck	Cat# SRP3069
Isopropanol	VWR	Cat# 20880
Knockout Serum Replacement (KOSR)	Thermo Fisher Scientific	Cat# 10828028
Lipidure	AMSbio	Cat# AMS.52000011GB1G
Matrigel Growth Factor Reduced (GFR) Basement Membrane Matrix	CORNING	Cat# 354230
mTeSR™ 1	Stem Cell Technologies	Cat# 85850
Methanol	VWR	Cat# 20846.326
N2 Supplement	Thermo Fisher Scientific	Cat# 17502001
Non-Essential AminoAcids	Thermo Fisher Scientific	Cat# 11140050
Normal Goat Serum	Millipore	Cat# S26-100ml
OCT embedding matrix	CellPath	Cat# KMA-0100-00A
Paraformaldehyde	Sigma-Aldrich	Cat# 158127
Phosphate Buffered Saline (PBS)	Thermo Fisher Scientific	Cat# 14190250
Penicillin/Streptomycin	Thermo Fisher Scientific	Cat# 15140130
Protease IV	ACD	Cat# 322336
Retinoic acid	Merck	Cat# R2625
ROCK inhibitor (Y-27632)	CHEMDEA	Cat# CD0141
T3	Merck	Cat# 709719
Taurine	Merck	Cat# T8691
Triton X-100	Sigma-Aldrich	Cat# T8787
Vectashield	Vector Laboratories	Cat# H-1000

**Critical commercial assays**		

Chromium Next GEM Chip G Single Cell kit	10x Genomics	Cat#1000120
Chromium Next GEM Chip H Single Cell Kit v1.1	10x Genomics	Cat#1000162
Chromium Next GEM Single Cell ATAC Library & Gel Bead Kit, version 1.1	10x Genomics	Cat#1000175
Chromium Next GEM Single Cell 3’ Kit, version 3.1	10x Genomics	Cat#1000268
Chromium Single Cell 3’ Library & Gel Bead Kit, version 3.1	10x Genomics	Cat#1000121
Neurosphere dissociation kit	Miltenyi Biotech	Cat#130-095-943; RRID:SCR_020290
Visium Spatial Gene Expression Kit, version 1	10x Genomics	Cat#1000184; RRID:SCR_023571
Visium Spatial Tissue Optimization Kit	10x Genomics	Cat#1000193

**Deposited data**		

GEO	GEO: GSE235577	
Zenodo	Zenodo: https://doi.org/10.5281/zenodo.10658110	

**Experimental models: Cell lines**		

hiPSCs (WT2)	Laboratory of Prof Majlinda Lako, Newcastle University	Melguizo-Sanchis et al.^56^, Buskin et al.^57^

**Oligonucleotides**		

Hs-ZIC1-C2	ACD	Cat#542991-C2
Hs-HES6-C3	ACD	Cat#521301-C3
Opal 520	Akoya Biosciences	Cat#FP1487001KT
Opal 650	Akoya Biosciences	Cat#FP1496001KT
Positive control probe	ACD	Cat#320861
Negative control probe	ACD	Cat#320871

**Software and algorithms**		

ZEN software	Zeiss, Germany	
Spaceranger (version 1.0)	10x Genomics	
Seurat (version 4.3.0)	https://satijalab.org/seurat/	RRID:SCR_016341
Monoclone 3 (version 1.3.1)	Bioconductor	
Cellranger ATAC software (version 1.2)	10x Genomics	
Bedtools merge (version 2.30)	Bedtools	RRID:SCR_006646
Cellranger (Version 3.01)	10x Genomics	
Ingenuity Pathway Analysis (IPA) software	Qiagen	RRID:SCR_008653
SCENIC+ (Version 1.0.1)	https://github.com/aertslab	
Python, scanpy (version 1.9.5)	https://github.com/scverse/scanpy	RRID:SCR_018139
DoubletFinder (Version 2.0.3)	Bioconductor	RRID:SCR_018771
Harmony (Version 0.1.1)	Bioconductor	RRID:SCR_022206
cell2Location (Version 0.13)	https://github.com/BayraktarLab/cell2location	
Spaniel (Version 1.12)	Bioconductor	
Signac (Version 1.6)	Bioconductor	RRID:SCR_021158
Chromvar (Version 1.20)	Bioconductor	
ComplexHeatmap (Version 2.14)	Bioconductor	RRID:SCR_017270

**Other**		

6 Well plate	Helena/TTP	Cat#92406
96-well plates (U-bottom)	Helena/TPP	Cat#92697T
6-well low attachment plates	CORNING	Cat#3471
Axio Observer.Z1	Zeiss, Germany	
Countess II FL Automated Cell Counter	Life Technologies	RRID:SCR_020236
Nikon ECLIPSE Ti inverted microscope	Nikon Instruments Inc., USA	RRID:SCR_021242
Zeiss Axio Imager.Z1 microscope	Zeiss, Germany	RRID:SCR_018876

### Resource availability

#### Lead contact

Further information and requests for resources and reagents should be directed to and will be fulfilled by the lead contact, Majlinda Lako (majlinda.lako@ncl.ac.uk). Request for bioinformatic pipelines and data analyses should be sent to rachel.queen@ncl.ac.uk.

#### Materials availability

This study did not generate unique reagents or cells.

#### Data and code availability

• Single cell RNA-Seq, single cell ATAC-Seq and ST data have been deposited to GEO under the following accession number: GEO_GSE235577 (https://www.ncbi.nlm.nih.gov/geo/query/acc.cgi?acc=GSE235577) and publicly available as of the 15^th^ February 2024.
• All original code has been deposited at Zenodo (https://doi.org/10.5281/zenodo.10658110) and is publicly available as of the 15^th^ February 2024.
• Any additional information required to reanalyze the data deposited in this paper is available from the lead contact upon request.

### Experimental model and study participant details

#### Pluripotent stem cells

Human iPSCs WT2 derived from an unaffected female patient and characterised in our group were used for this study.^56^^,^^57^

### Method details

#### Retinal organoid differentiation

hiPSCs (WT2) were expanded in mTESR™1 (StemCell Technologies, 05850) on growth factor reduced Matrigel coated plates (BD Biosciences, San Jose, CA) at 37°C and 5% CO_2_. For the generation of ROs, confluent hiPSCs were dissociated into single cells using Accutase (Gibco, A1110501) and were seeded at a density of 7,000 cells/well onto Lipidure (AMSbio, AMS.52000011GB1G) pre-coated 96-well plates (U-bottom, Helena, 92697T) in mTeSR™1 supplemented with 10 μM Y-27632 ROCK inhibitor (Chemdea, CD0141). 200 μl of differentiation medium was added after 2 days and hereupon half of the differentiation medium was changed every 2 days until day 18 of differentiation.^58^ After day 18, the differentiation media was supplemented with Fetal Calf Serum (FCS; Thermo Fisher), Taurine (Merck) and T3 (Merck) and ROs were further cultured in 6-well low attachment plates (Corning, 3471). Retinoic Acid (RA; Merck) was added from day 90 to day 120 of differentiation. The media was changed every 2-3 days for remaining differentiation. Different media compositions are shown in Table S10.

Prior to ROs collection, brightfield images were taken using a AxioVert upright microscope (Zeiss, Germany). ROs were collected at different developmental stages for experiments, starting with early developmental samples (day 10, 20 and 35), followed by the mid developmental (day 45, 60 and 90) and late developmental (day150 and 210) samples.

#### scRNA- and -ATAC-Seq

ROs samples of different developmental stages (day 10, 20, 35, 45, 60, 90, 150 and 210) were dissociated to single cells using a neurosphere dissociation kit (Miltenyi Biotech, Cat number 130-095-943) (Tables S1, S3, and S5). Briefly, 96-144 ROs (age dependent) were collected, washed with Hanks' Balanced Salt Solution (HBSS; Thermo Fisher, J67763-AP) and incubated for 10 minutes at 37°C under slow, continuous rotation in Buffer/Enzyme solution prepared according to manufacturer’s instructions. Organoids were than manually dissociated using a wide-tipped Pasteur pipette by pipetting up and down. After another 5 minutes incubation at 37°C, organoids were further dissociated by pipetting up and down until a single-cell suspension was achieved. The single-cell suspension was applied to a Pre-Separation Filter followed by a HBSS wash. The filtered single-cell suspension was centrifuged at 300xg for 10 minutes, the supernatant was aspirated, and the cell pellet was resuspended in an appropriate amount of HBBS solution containing 1% FBS (Miltenyi Biotec, 130-091-376). Cells were then counted (Countess II FL Automated Cell Counter, Invitrogen) and 10,000 cells from each sample were captured using Chromium Next GEM Chip G Single Cell kit (10x Genomics, 1000120) and further processed according to manufacturer’s manual (Chromium Next GEM Single Cell 3’ Kit, version 3.1, 10x Genomics, 1000268). Sequencing libraries were generated using the Chromium Single Cell 3’ Library & Gel Bead Kit (version 3.1, 10x Genomics, 1000121) for scRNA-Seq. For scATAC-Seq 10,000 of the subsequent nuclei were captured (Chromium Next GEM Chip H Single Cell Kit v1.1, 10x Genomics, 1000162), and sequencing libraries generated using the Chromium Next GEM Single Cell ATAC Library & Gel Bead Kit (version 1.1, 10x Genomics, 1000175). Single cell RNA-Seq libraries were sequenced to 50,000 reads per cell and scATAC-Seq libraries were sequenced to 25,000 reads per nucleus on an Illumina NovaSeq 6000.

#### scRNA-Seq analysis

Cellranger mkfastq version 3.01 was used to de-multiplex the BCL files into FASTQ files. Reads were then aligned and quantified using Cellranger count and the human reference genome GRCh38. The quality of the cells in each sample were checked in R. Cells which had fewer than 1000 reads or 500 genes or more than 10% mitochondrial reads were removed from downstream analysis. DoubletFinder was used to identify doublets within the data these were then removed from the datasets. Each sample was assessed individually following the Seurat (version 4.3.0). The standard clustering workflow was followed. This included normalisation to remove cell to cell differences, scaling the data to remove differences in expression levels between genes, identification of a set of 2000 biologically informative highly variable genes and principle component reduction of data, and clustering of the data. The following factors were regressed out during the scaling process, "percent.mt", "nCount_RNA", "nFeature_RNA".

Retinal cells were then selected from the individual samples and batch effects removed by Harmony (version 0.1.1) to create an integrated dataset. The data was visualised using a Uniform Manifold Approximation and Projection (UMAP) based on the first 10 batch corrected coordinates and clusters identified using Seurat. Differentially expressed markers between each cluster were identified using the Seurat FindMarkers function with the method Wilcoxon test (Tables S1 and S3). Cluster identity was performed based on expression of well-known retinal cell type specific markers (Table S11).

The combined dataset was subset to study development trajectory within the following cell types: pCM-RPC-T1-T2-T3, pCM-RPC-T1-T2-HC-AC-RGC, pCM-RPC-T1-T3-Cones-Rods-BC (Table S4). Cells within each of these groups were selected and then the data was re-clustered using the method described for the full dataset. We then reassessed the annotations to ensure that we had high confidence in the cell type assignment. Pseudotime trajectories for each developmental branch were created using Monocle 3. Genes which were differentially expressed between cell types were identified within the subsets. These were visualised in heatmaps where cells were ordered within cell type by pseudotime.

#### scATAC-ATAC analysis

Cellranger ATAC software (version 1.2) was used to identify peaks in each of the samples. Bedtools merge (version 2.30) was used to create shared peaks and the data was reanalysed using Cellranger ATAC reanalyse. Quality control steps were performed using Signac. The datasets were imported using Signac and quality control steps were performed to remove cells low quality cells. We filtered cells which had fewer than 20% of reads in peak region fragments, or less than 3000 peak region fragments. Cells with a TSS enrichment score of less than 2 and Blacklist ration greater than 0.05 or a nucleosome signal of less than 4 were also removed from downstream analysis.

The standard Signac workflow to cluster the cells, which consists of term frequency-inverse document frequency (TF-IDF) normalisation; singular value decomposition (SVD) dimension reduction; followed by UMAP reduction was applied to the data. Seurat was then used to cluster the cells. LSI components 2 to 30 were used to create the UMAP and for clustering analysis. A gene activity matrix was generated using Signac. This was used to predict upregulated genes in each cluster and assign the clusters to cell types (Table S5).

A combined dataset was created by selecting retinal cells from each sample. Retinal cell types were selected from each sample and a combined dataset was created using the method described in the previous paragraph. The Logistic regression (LR) test from the FindAllMarkers function was used to identify differentially accessible peaks for each of the annotated cell types (Table S6). The ComplexHeatmap package was used to plot the average peak value for each differentially accessible peak for each cell type.

The per cell motif activity was computed using Chromvar. Enriched motifs for each cell type were identified using FindAllMarkers. ComplexHeatmap was used to show the top cell type enriched motifs ordered by average difference in z-score (Table S7). Signac was used to generate motif plots.

#### Network analysis of scRNA-Seq and scATAC-Seq data

Using Qiagen Ingenuity Pathway Analysis (IPA) software the differentially expressed gene lists identified in RNAseq were analysed using IPA core analysis. IPA includes manually curated knowledge which integrates diverse biological data sources, including gene expression, molecular interactions, and pathway information from a large experimental database to identify significant biological pathways. Using the “predict upstream regulators” function we looked for upstream regulators which would explain the gene expression patterns observed. The DA peaks and motif predictions obtained in our ATAC Seq data were then overlaid onto the networks to look for a consensus in the predictions. (Table S8).

We then went onto to study the enhancer and gene regulatory interactions within our scRNA-Seq and scATAC-Seq data using SCENIC+ (version 1.0.1). Firstly, using pycisTopic (version 1.0.3) we identified and binarized cis-regulatory topics from our integrated ATAC dataset. Next, pycistarget (version 1.0.3) was then used to identify enriched motifs. We exported the integrated scRNA data into python using scanpy (version 1.9.5) and then combined the create a joint RNA/ATAC object using SCENICplus, using the setting “multi_ome_mode = False”. The “run_scenicplus” wrapper was then run to infer enhancer driven GRNs (eGRNs). The regulon_specificity_scores (RSS) for each cell type was calculated and these scores were used to show the eGRN specificity for each cell type as a heatmap. We plotted selected eGRN. The CRX and NEUROD1 were very large and were filtered to only include highly variable genes and the filtered eRegulons.

#### ST

RO samples were collected and fresh frozen at day 10, 20, 35, 45, 60, 90, 150 and 210 of differentiation (Table S2). Each sample contained 10-40 retinal organoids, depending on the stage of differentiation. Sections (10μm) were cut (Leica Cm1860) and carefully placed into the capture areas of the spatial transcriptomic slides. Slides were stored at -80°C until needed. Spatial transcriptome analyses were performed with the Visium Spatial Gene Expression Kit (version 1, 10x Genomics, 1000184). First, the tissue optimisation was performed defining 24-30 minutes as the most optimal permeabilization time window (Visium Spatial Tissue Optimization Kit, 10x Genomics, 1000193). The gene expression ST procedure was performed according to manufacturer’s instructions. Briefly, sections in the four capture areas for each sample were fixed, haematoxylin and eosin (H&E) stained according to manufacturer’s instructions (Visium Spatial Gene Expression kit; 10x Genomics). For this, sections were fixed using ice-cold methanol (VWR, 20846.326), incubated in isopropanol (VWR, 20880) and air dried. After the Hematoxylin (Dako, S3309) incubation, several washing steps, incubation with Bluing Buffer (Dako, CS702) and further washing steps, the sections were incubated with an Eosin solution (Sigma-Aldrich, HT110216). Subsequently, washing steps were carried out and sections were then air dried and imaged to preserve histological information using a NikonTiE inverted microscope at 10x magnification (Plan Fluor 10x 0.3 NA). Image tiles captured and stitched together using Nikon Elements software, enabling to overlay the cell tissue image and the gene expression data later. After permeabilization, reverse transcription reagents were added on top of the tissue sections. The tissue sections were subsequently removed, leaving the cDNA coupled to the arrayed oligonucleotides on the slide. Then the cDNA-RNA hybrids were cleaved off the chip and the sequencing libraries were prepared. The sequencing depth varied between 100,000,000 and 250,000,000 million reads.

#### ST analysis

The data was de-multiplexed, aligned and quantified using Spaceranger version 1.0. The data was aligned to the human reference genome GRCh38. Spaceranger generated spot co-ordinates calculated using fiducial detection, and regions under the tissue section. Spaniel (version 1.12) was used to import the data into R. The 4 consecutive sections tissue sections from each sample were clustered using the Seurat scRNA-Seq pipeline described above. A resolution of 0.5 was used for the cluster analysis. Differentially expressed genes were identified between clusters. The spatial expression and cluster plots were generated using Spaniel. Cluster identity was defined based on expression of well-known retinal cell markers (Table S11). We then estimated the number of the cells under each spot from the original H & E images we counted the cell numbers in a in region 55um in diameter from 3 different organoids, and two locations (apical region and central region) for each timepoint and then calculated the average cell number/spot for each timepoint (Table S12).

To understand the composition of each spot we used cell2Location to establish a spatial mapping of cell types. The spatial spot data from D90, D150, D210 each were decomposed into single cell using reference signatures from the corresponding scRNA-seq datasets. The abundance estimates for each cell type are shown in Figure S12.

#### RNAscope

RNAscope *in situ* hybridization assay was used to determine the expression profile of *ZIC1* and *HES6* during human retinal organoid development. Fresh frozen sections of 15-20 ROs aged day 45 as used for spatial transcriptomic experiments were formalin fixed for 1 h at 4°C. The sections were dehydrated through a series of ethanol 50%, 70% and two changes of 100%. The sections were incubated with a protease IV (ACD- Cat. No. 322336) for 15 min at 40°C. RNAscope probes Hs-ZIC1-C2 (ACD- Cat No. 542991-C2) and Hs-HES6-C3 (ACD- Cat No. 521301-C3) were hybridised to the tissue for 2 h at 40°C followed by multiple rounds of signal amplification. Positive (ACD- Cat No. 320861) and negative (ACD- Cat No. 320871) control probes were used to confirm specificity. The annealed probes were detected using Opal fluorophores OPAL 650 (C2) and OPAL 520 (C3) and imaged using an Axio Imager upright microscope with Apotome structured illumination fluorescence and the ZEN software.

#### IF analyses

10-15 ‘Eye-like’ ROs were collected at day 45 and day 90 of differentiation. After fixation using 4% paraformaldehyde (PFA; Sigma-Aldrich, 158127) organoids were cryopreserved, embedded in OCT (Cell Path Ltd, Newtown, UK) and sectioned (10 μm) on a cryostat (Leica Cm1860). IF was performed as previously described.^59^ Briefly, sections were blocked for 1h at room temperature using Phosphate buffered Saline (PBS) containing 10% Normal Goat Serum (NGS, Millipore, S26) and 0.3% Triton X-100 (Sigma-Aldrich, T8787). Primary antibodies were appropriate diluted in PBS containing 0.1% Bovine serum albumin (BSA; Sigma-Aldrich, A2153) and 0.1% Triton-X-100. Sections were reacted against the following primary antibodies: CRYAA (1:50; gift from Roy Quinlan), CRYAB (1:500, Abcam, ab76467), CRX (1:200, Abnova, H00001406-MO2), Ki67 (1:200, Abcam, ab15580), KRT13 (1:500, Abcam, ab52816), KRT15 (1:500, Abcam, ab92551), Lumican (1:50, Santa Cruz Biotechnologies, sc-166871), MT2A (1:100, Sigma-Aldrich, SAB1402848) and NP63 (1:250, Abcam, ab735). After an overnight incubation and several washing steps in PBS, secondary antibodies, conjugated to Alexa488 (Jackson Immuno Research Laboratories) and Cy3 (Jackson Immuno Research Laboratories) were diluted in PBS and applied for 2h at room temperature. Following several washing steps in PBS, sections were embedded in Vectashield (Vector Laboratories, H-1000) containing Hoechst (1:1000; Thermo Fisher, H3570) and stored appropriately before imaging. Antibody specificity was assessed by omitting the primary antibodies. Images were obtained using a Zeiss Axio Imager.Z1 microscope with ApoTome.2 accessory equipment and Zen software. Representative images are displayed as a maximum projection and adjusted for brightness and contrast in Adobe Photoshop CS6 (Adobe Systems).

#### Comparison of organoid samples with human retina

All organoid ATAC-seq samples were compared with 12 ATAC-seq samples of developing human eyes and neural retina from 10-21 post-conception weeks (GSE234003).^24^ A unified set of overlapping peak ranges from both datasets were created. Peaks were filtered if they were greater than 10000bp or less than 20bp. Cellranger-atac was used to call peaks in all organoid and human retina samples using the shared ranges. The data was then processed following the Signac workflow described above. Harmony was used to batch differences between the human fetal retina and organoid samples. The data was clustered at a resolution of 0.8 and 36 clusters were identified and visualised on a UMAP plot. These were annotated as cell types using the gene activity values (Table S9). Monocle 3 was then used to create a pseudotime trajectory. The median pseudotime values for each sample were then plotted against age in days. Beeswarm plots were used to compare the pseudotime range of organoid and human cells for each cell type.

### Quantification and statistical analysis

There were no quantification and statistical analyses used other than those described in the single cell analyses sections.

## Supplemental information

Document S1. Figures S1–S12 and Tables S10 and S12

Table S1. scRNA-Seq analysis of ROs at day 10–210 of differentiation, related to Figures 1, 2, and 3 Cluster annotations and highly expressed genes for each cluster and are shown on separate spreadsheets for each sample.

Table S2. Spatial transcriptomic analysis of ROs at day 10–210, related to Figures 1, 2, and 3 Cluster annotations and highly expressed genes for each cluster and are shown on separate spreadsheets for each sample.

Table S3. scRNA-Seq analysis of eye-like organoids at day 45 and day 90 of differentiation, related to Figures 1 and 2 Cluster annotations and highly expressed genes for each cluster and are shown on separate spreadsheets for each sample.

Table S4. Integrated scRNA-Seq analysis of ROs (day 10–210 of differentiation) and pseudotime analysis, related to Figures 4, 5, and 6 The first spreadsheet shows the integrated scRNA-Seq data for ROs (day 10–210 of differentiation). The second spreadsheet displays highly expressed markers characterizing lineage transitions from pCM, RPCs to T1, T2, T3. The third spreadsheet shows highly expressed markers characterizing lineage transitions from pCM, RPCs, T1 to T3, photoreceptors and bipolar cells. The fourth spreadsheet shows highly expressed markers characterizing lineage transitions from pCM, RPCs to T1, T2, RGCs, horizontal and amacrine cells.

Table S5. scATAC-Seq analysis of ROs at day 10–210 of differentiation, related to Figures 7 and 8 Gene activity estimates were generated using Signac. Genes with high activity scores for each cell type are shown on separate spreadsheets for each sample. The final spreadsheet shows the gene activity scores for the integrated datasets. All retinal cells across samples were integrated using harmony and the data was then re-clustered to produce integrated results.

Table S6. Differential accessibility peaks for each retinal cell type using the integrated scATAC-Seq dataset, related to Figures 7 and 8 Peaks were linked to genes using Cellranger and classified as either promoter, distal or intergenic. The distance from the gene is shown for each peak. Abbreviations: eRPCs – early RPCs, lRPCs – late RPCs, HCs-horizontal cells, ACs-amacrine cells, BCs-bipolar cells, pCM – putative ciliary margin, MCs – Muller glia cells, RGCs-retinal ganglion cells, T1, T3 – transient neurogenic progenitors.

Table S7. Predicted transcription factor binding (motifs) for each cell type in ROs using the integrated scATAC-Seq dataset, related to Figures 7 and 8 ChromVAR was used to characterize transcription factor motifs for each cell type. Abbreviations: eRPCs – early RPCs, lRPCs – late RPCs, HCs-horizontal cells, ACs-amacrine cells, BCs-bipolar cells, pCM – putative ciliary margin, MCs – Muller glia cells, RGCs-retinal ganglion cells. T1, T3 – transient neurogenic progenitors.

Table S8. List of significant gene expression regulators in RPCs, transient neurogenic progenitors (T1, T2), photoreceptors, bipolar, horizontal and amacrine cells, related to Figures 7 and 8

Table S9. Integrated dataset from scATAC-Seq of ROs (day 10–210 of differentiation) and human developing retina (pcw 10–21), related to Figures 7 and 8 Peaks were linked to genes using Cellranger and further processed with Signac. Harmony was used to reduce batch differences between ROs and human developing retina samples.

Table S11. A summary of all retinal cell type specific markers used for cluster identification in single cell analyses, related to Figures 1, 2, 3, 7, and 8
