# Supplementary material for: Deciphering the spatiotemporal transcriptional and chromatin accessibility of human retinal organoid development at the single-cell level
Source: iScience. 2024 Mar 4;27(4):109397. doi: 10.1016/j.isci.2024.109397 (PMC10952046; doi:10.1016/j.isci.2024.109397)
Supplement: Document S1. Figures S1–S12 and Tables S10 and S12 [file mmc1.pdf]

iScience, Volume *27*

Supplemental information

Deciphering the spatiotemporal transcriptional and chromatin accessibility of human retinal organoid development at the single-cell level

Birthe Dorgau, Joseph Collin, Agata Rozanska, Veronika Boczonadi, Marina Moya-Molina, Adrienne Unsworth, Rafiqul Hussain, Jonathan Coxhead, Tamil Dhanaseelan, Lyle Armstrong, Rachel Queen, and Majlinda Lako

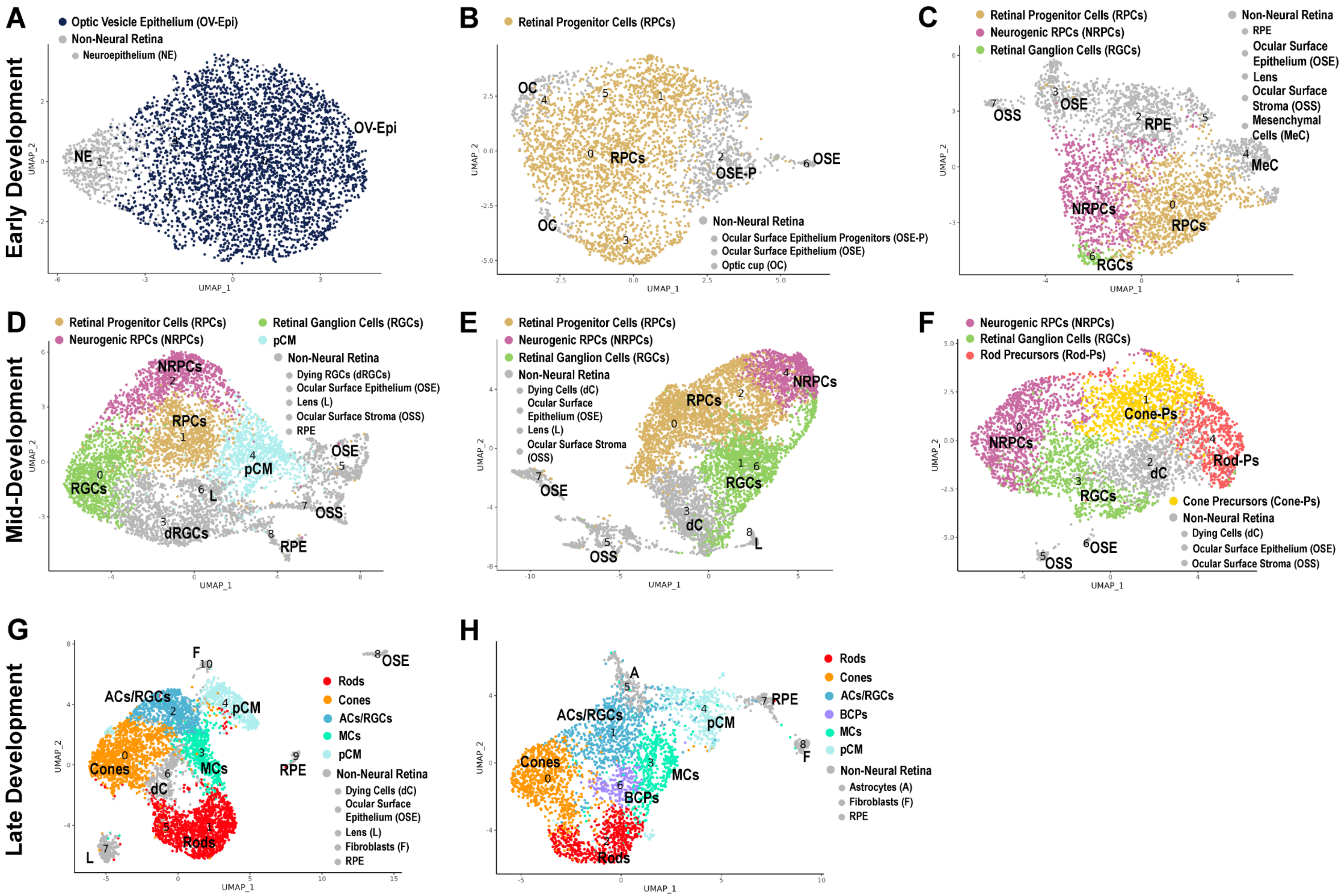
Early Development
Mid-Development
Late Development
A
Optic Vesicle Epithelium (OV-Epi)
Non-Neural Retina
Neuroepithelium (NE)
NE
OV-Epi
B
Retinal Progenitor Cells (RPCs)
OC
RPCs
OSE-P
OSE
Non-Neural Retina
Ocular Surface Epithelium Progenitors (OSE-P)
Ocular Surface Epithelium (OSE)
Optic cup (OC)
C
Retinal Progenitor Cells (RPCs)
Neurogenic RPCs (NRPCs)
Retinal Ganglion Cells (RGCs)
Non-Neural Retina
RPE
Ocular Surface Epithelium (OSE)
Lens
Ocular Surface Stroma (OSS)
Mesenchymal Cells (MeC)
OSS
OSE
RPE
MeC
NRPCs
RPCs
RGCs
D
Retinal Progenitor Cells (RPCs)
Neurogenic RPCs (NRPCs)
Retinal Ganglion Cells (RGCs)
pCM
Non-Neural Retina
Dying RGCs (dRGCs)
Ocular Surface Epithelium (OSE)
Lens (L)
Ocular Surface Stroma (OSS)
RPE
NRPCs
RPCs
RGCs
pCM
OSE
L
OSS
dRGCs
RPE
E
Retinal Progenitor Cells (RPCs)
Neurogenic RPCs (NRPCs)
Retinal Ganglion Cells (RGCs)
Non-Neural Retina
Dying Cells (dC)
Ocular Surface Epithelium (OSE)
Lens (L)
Ocular Surface Stroma (OSS)
RPCs
NRPCs
RGCs
OSE
dC
L
OSS
F
Neurogenic RPCs (NRPCs)
Retinal Ganglion Cells (RGCs)
Rod Precursors (Rod-Ps)
Cone Precursors (Cone-Ps)
Non-Neural Retina
Dying Cells (dC)
Ocular Surface Epithelium (OSE)
Ocular Surface Stroma (OSS)
Cone-Ps
NRPCs
Rod-Ps
dC
RGCs
OSE
OSS
G
Rods
Cones
ACs/RGCs
MCs
pCM
Non-Neural Retina
Dying Cells (dC)
Ocular Surface Epithelium (OSE)
Lens (L)
Fibroblasts (F)
RPE
F
OSE
ACs/RGCs
pCM
Cones
MCs
dC
RPE
Rods
L
H
Rods
Cones
ACs/RGCs
BCPs
MCs
pCM
Non-Neural Retina
Astrocytes (A)
Fibroblasts (F)
RPE
A
RPE
ACs/RGCs
pCM
Cones
MCs
BCPs
F
Rods
UMAP_1
UMAP_2

Figure S1: Cell clusters identified in ST analyses in early, mid, and late retinal development (related to Figures 1-3 and Table S2). **A-H)** ST UMAP plots showing individual clusters during early RO differentiation, including day 10 (**A**), day 20 (**B**) and day 35 (**C**), mid retinal development, composed of day 45 (**D**), day 60 (**E**) and day 90 (**F**) and late retinal development with day 150 (**G**) and day 210 (**H**). Cluster annotations and highly expressed genes for each cluster are shown in Table S2.

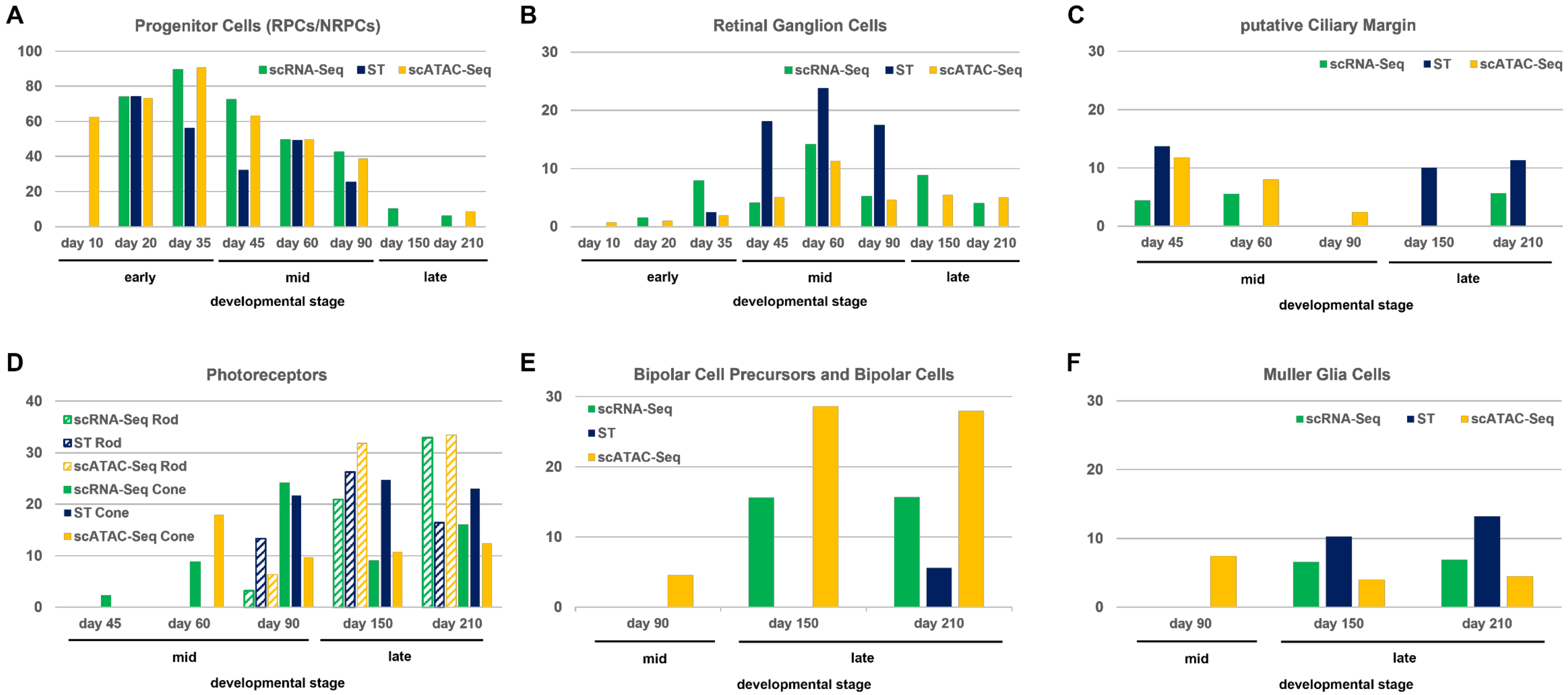

Figure S2: ScRNA-Seq, scATAC-seq and ST analyses comparison of retinal cell type development during RO differentiation (related to Figures 1-3 and 7-8). **A)** Progenitor cell populations (RPCs/NRPCs) peak at day 20 in ST data and day 35 in scRNA-Seq data and then decreases over time. **B)** RGCs population increases up to day 60 of differentiation and diminishes afterwards. Note RGCs were excluded at day 150 and 210 differentiation from ST analyses as they were detected in a mixed cluster with amacrine cells. **C)** The putative Ciliary Margin cluster was first observed at day 45. **D)** Photoreceptors (cones and rods) expand from day 45 onwards during RO development, even so cones emerge earlier than rods. **E)** Bipolar cells emerge from day 90 in scATAC-Seq and day 150 onwards in scRNA-Seq analysis, revealing a population increase until day 210: those were first detected at day 210 of differentiation in ST analysis. **E)** Muller glia cells were detected from day 90 onwards in scATAC-Seq data and from day 150 onwards in scRNA-Seq and ST datasets.

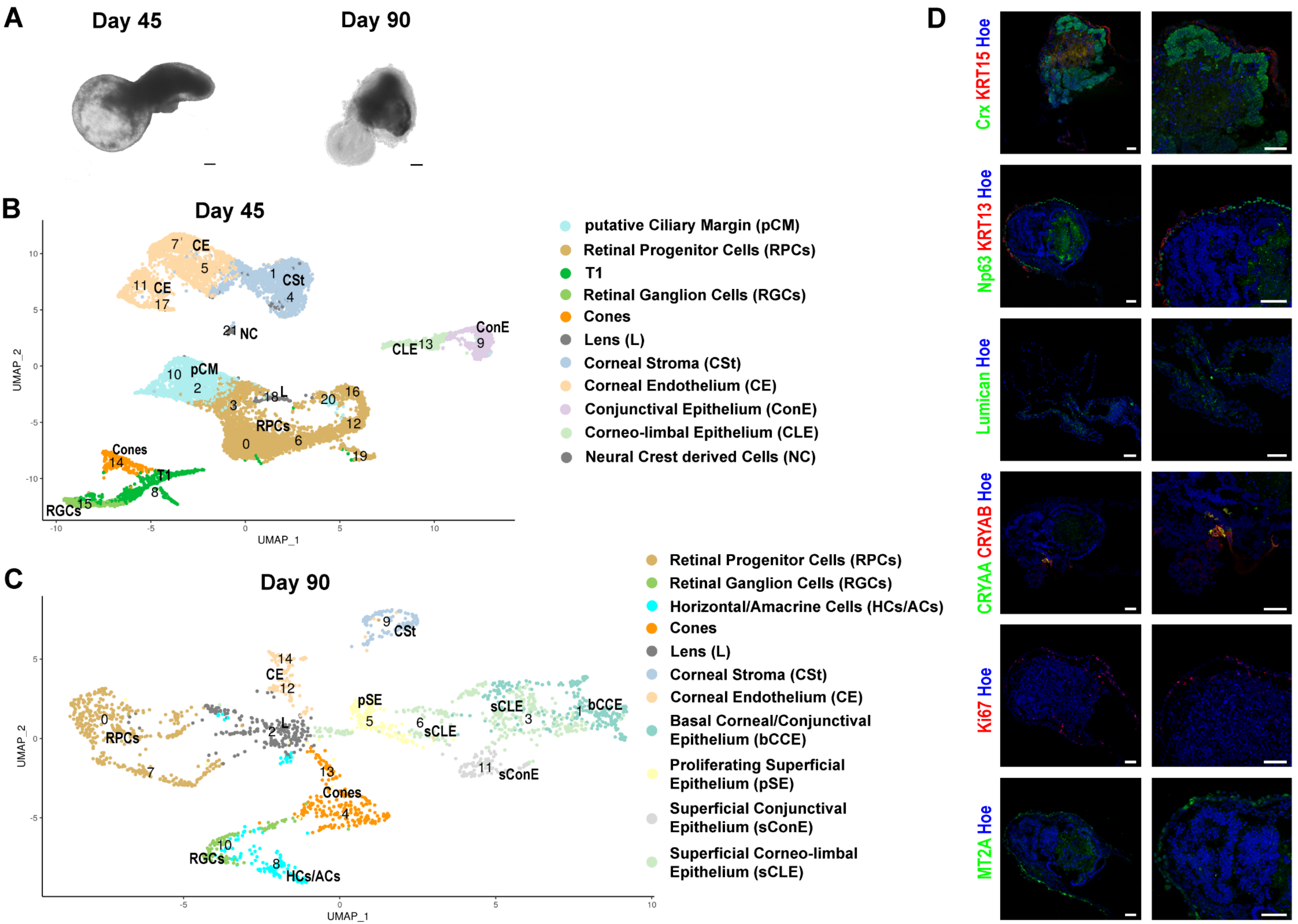

A
Day 45
Day 90
B
Day 45
putative Ciliary Margin (pCM)
Retinal Progenitor Cells (RPCs)
T1
Retinal Ganglion Cells (RGCs)
Cones
Lens (L)
Corneal Stroma (CSt)
Corneal Endothelium (CE)
Conjunctival Epithelium (ConE)
Corneo-limbal Epithelium (CLE)
Neural Crest derived Cells (NC)
UMAP_1
UMAP_2
C
Day 90
Retinal Progenitor Cells (RPCs)
Retinal Ganglion Cells (RGCs)
Horizontal/Amacrine Cells (HCs/ACs)
Cones
Lens (L)
Corneal Stroma (CSt)
Corneal Endothelium (CE)
Basal Corneal/Conjunctival Epithelium (bCCE)
Proliferating Superficial Epithelium (pSE)
Superficial Conjunctival Epithelium (sConE)
Superficial Corneo-limbal Epithelium (sCLE)
D
Crx KRT15 Hoe
Np63 KRT13 Hoe
Lumican Hoe
CRYAA CRYAB Hoe
Ki67 Hoe
MT2A Hoe

Figure S3: Emergence of eye-like organoids at the mid stages of organoid differentiation (related to Figures 1, 2 and Table S3). **A)** Morphology of eye-like organoids at day 45 and day 90. Scale bars, 100 μm. **B, C)** scRNA-Seq UMAP plots of eye-like organoid cells at day 45 (**B**) and day 90 (**C**) revealing a retinal core with RPCs, T1 transient neurogenic progenitors, RGCs and cones alongside lens and cornea which displays the formation of all layers: the endothelium, the stroma, and the epithelium. Cluster annotations and highly expressed genes for each cluster are shown in Table S3. **D)** IF analysis of eye-like organoids at day 45 of differentiation, confirming the presence of a retinal core by Crx-positive cells (green) together with the presence of lens (CRYAA, green and CRYAB, red), limbal-corneal epithelium (KRT15, red), conjunctival epithelium (KRT13, red), corneal stroma cells (Lumican, green) and proliferative epithelial progenitor cells, identified by the expression of Np63 (green), MT2A (green) and Ki67 (red). Cell nuclei were counterstained with Hoechst. Scale bars, 20 μm. 144 ROs were used for day 45 and 96 ROs for day 90 in the scRNA-Seq analysis, which was performed once at each time point.

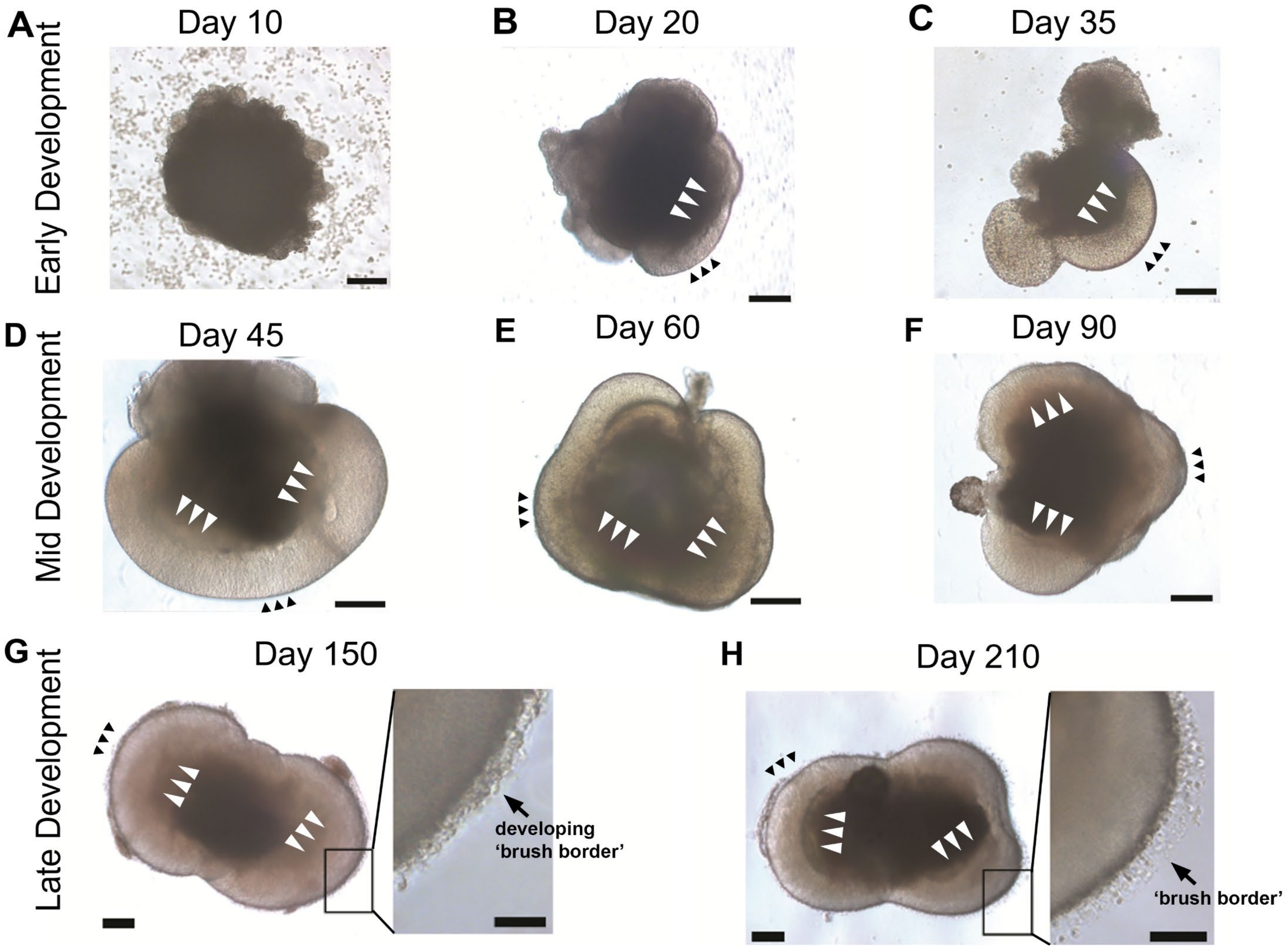
A
Day 10
Early Development
B
Day 20
C
Day 35
D
Day 45
Mid Development
E
Day 60
F
Day 90
G
Day 150
Late Development
developing
'brush border'
H
Day 210
'brush border'

Figure S4: Morphological development of ROs during the course of differentiation (related to Figures 1-3). **A-C)** Representative brightfield images of ROs at day 10 (**A**), day 20 (**B**) and day 35 (**C**) during early differentiation, revealing the formation of neuroepithelium at the apical edge of ROs from day 20 (**B**) onwards. **D-F)** ROs in mid developmental stages, day 45 (**D**), day 60 (**E**) and day 90 (**F**) of differentiation indicate the thickening of the neuroepithelium at the apical edge of organoids. **G, H)** ROs at day 150 (**G**) and day 210 (**H**) mature during late development, forming inner/outer segments ('brush border', black arrows, higher magnifications) at the apical edge of organoids. White arrowheads represent the center (basal site) of ROs and black arrowheads highlight the apical edge of ROs. Scale bars, 100 μm and 50 μm for higher magnifications.

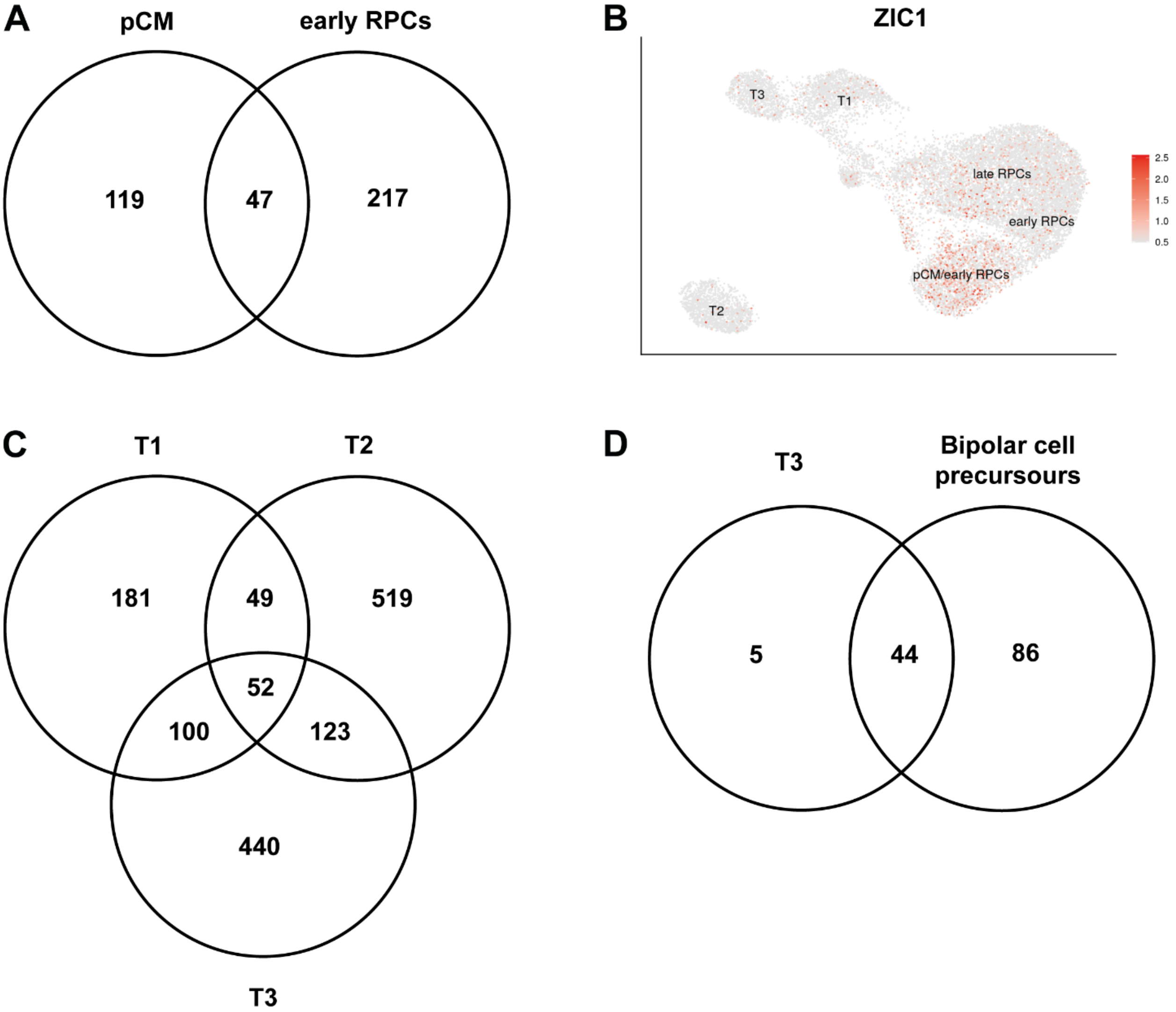

Figure S5: Gene expression profile comparison between the pCM and early RPCs (A), neurogenic transient populations (C) and neurogenic transient population T3 with bipolar cells (D) indicate overlapping as well as unique marker genes for cell type (related to Figure 4). **A)** Overlapping gene expression pattern of pCM and early RPCs, indicating some transcriptional overlap for the highly expressed genes in both clusters. **B)** *ZIC1* expression profile in the pCM, RPCs, and transient neurogenic progenitors (T1-T3) pseudotime trajectory showed highest expression in the pCM of ROs. **C)** Gene expression comparison between transient neurogenic progenitors, T1 - T3, revealing some transcriptional overlap for the highly expressed genes in these clusters. **D)** BCPs indicated a high transcriptional similarity to transient neurogenic progenitor population, T3, suggesting the lack of mature BCs in ROs. BCPs- bipolar cell progenitors, BCs- bipolar cells.

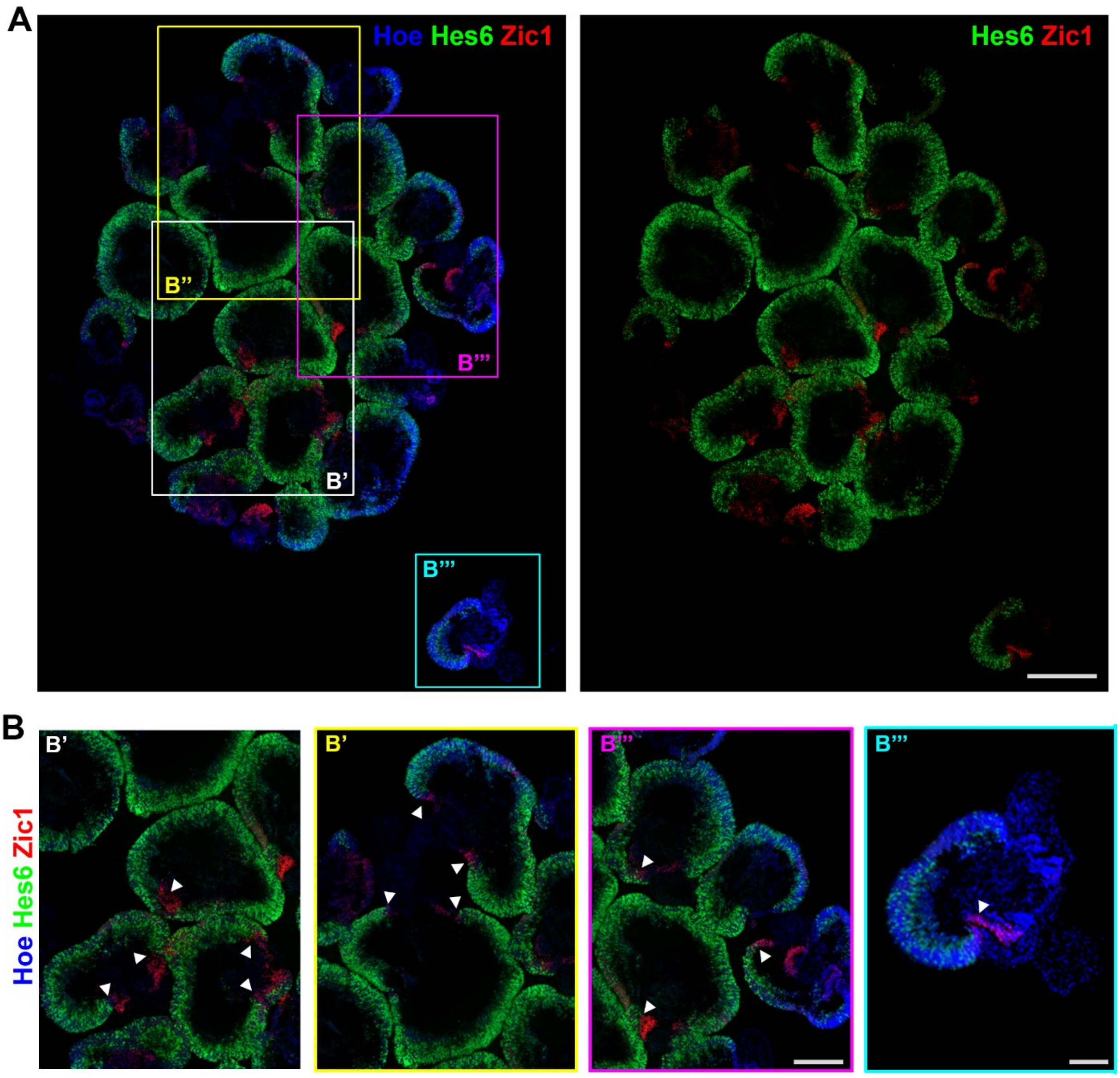

Figure S6: RNAscope reveals strong expression of early RPC marker *ZIC1*, in the pCM at day 45 of RO differentiation, while the late neurogenic RPC marker HES6 expression marks the rest of retinal neuroepithelium in a complementary pattern to *ZIC1* (related to Figure 4). **A)** Expression of *HES6* (green) and *ZIC1* (red) in ROs at day 45 of differentiation, confirming restricted expression of *ZIC1* in pCM. **B)** Higher magnification images showed the restricted *ZIC1* expression in the pCM of ROs and its spatial location at the distal tip of the retinal-like retinal structure (white arrowheads). Cell nuclei are counterstained Hoechst (Hoe, blue). Scale bars, 50 μm for A and 10 μm for B**.**

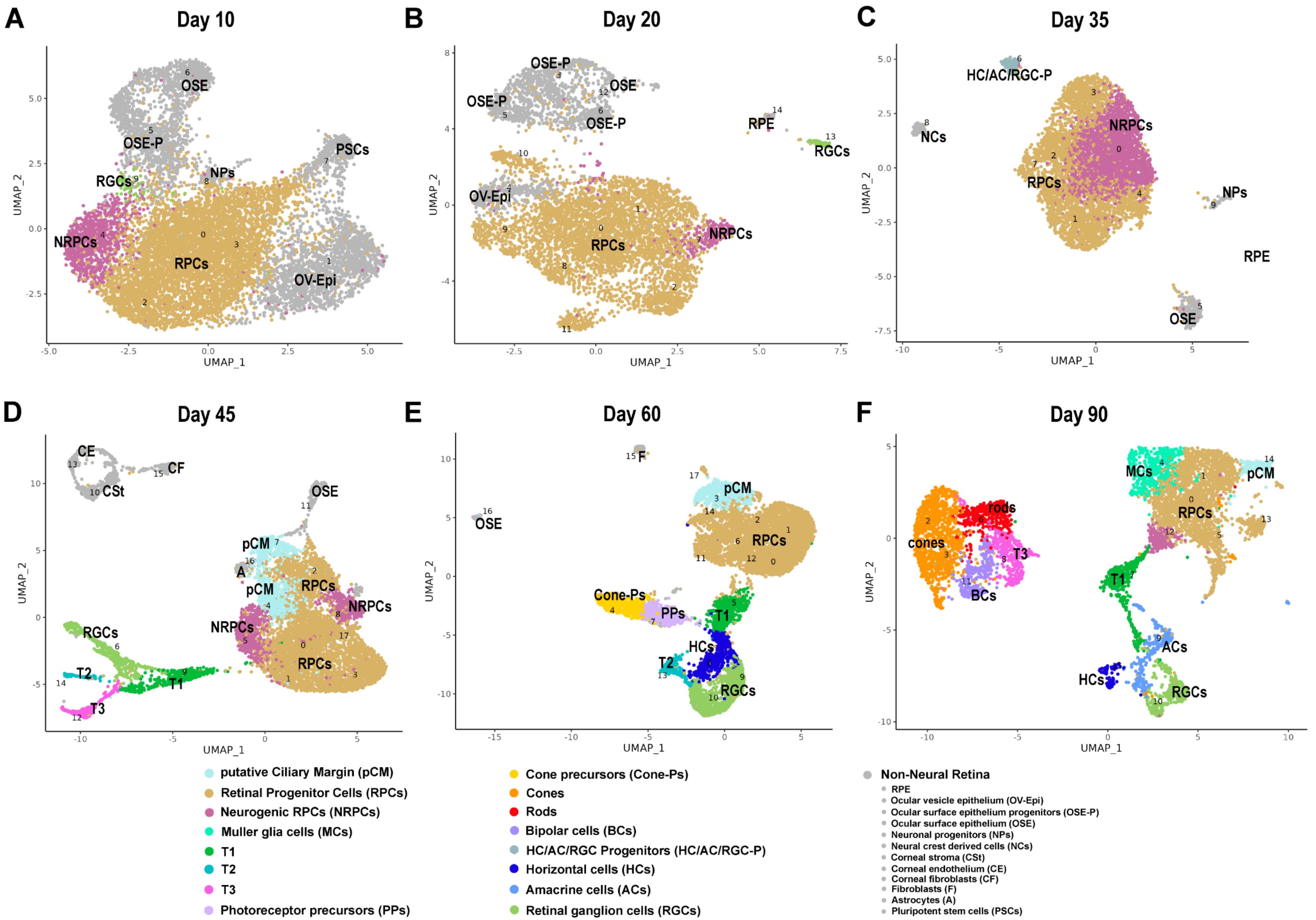

A
Day 10
OSE
OSE-P
RGCs
NPs
PSCs
NRPCs
RPCs
OV-Epi
UMAP_1
UMAP_2
B
Day 20
OSE-P
OSE
RPE
RGCs
OV-Epi
RPCs
NRPCs
C
Day 35
HC/AC/RGC-P
NCs
NRPCs
RPCs
NPs
RPE
OSE
D
Day 45
CE
CF
CSt
OSE
pCM
A
RPCs
NRPCs
RGCs
T2
T1
T3
E
Day 60
F
OSE
pCM
RPCs
Cone-Ps
PPs
T1
HCs
T2
RGCs
F
Day 90
MCs
pCM
rods
cones
T3
BCs
T1
ACs
HCs

putative Ciliary Margin (pCM)
Retinal Progenitor Cells (RPCs)
Neurogenic RPCs (NRPCs)
Muller glia cells (MCs)
T1
T2
T3
Photoreceptor precursors (PPs)
Cone precursors (Cone-Ps)
Cones
Rods
Bipolar cells (BCs)
HC/AC/RGC Progenitors (HC/AC/RGC-P)
Horizontal cells (HCs)
Amacrine cells (ACs)
Retinal ganglion cells (RGCs)
Non-Neural Retina
RPE
Ocular vesicle epithelium (OV-Epi)
Ocular surface epithelium progenitors (OSE-P)
Ocular surface epithelium (OSE)
Neuronal progenitors (NPs)
Neural crest derived cells (NCs)
Corneal stroma (CSt)
Corneal endothelium (CE)
Corneal fibroblasts (CF)
Fibroblasts (F)
Astrocytes (A)
Pluripotent stem cells (PSCs)

Figure S7: scATAC-Seq of ROs during early (day 10 - 35) and mid (day 45 - 90) developmental stages (related to Figures 7, 8 and Table S5). **A-C)** UMAP plots of scATAC-Seq of ROs at day 10 (**A**), day 20 (**B**) and day 35 (**C**) of differentiation. **D-E)** scATAC-Seq UMAP plots of ROs at day 45 (**D**), day 60 (**E**) and day 90 (**F**). Cluster identity defined on the basis on gene activity scores, calculated on open regions of the chromatin of retinal specific cell markers (Table S5). 144 ROs were used at day 10/20 and 96 ROs were used for day 35, 45, 60 and 90. ScATAC-Seq was performed once for each time point of RO differentiation.

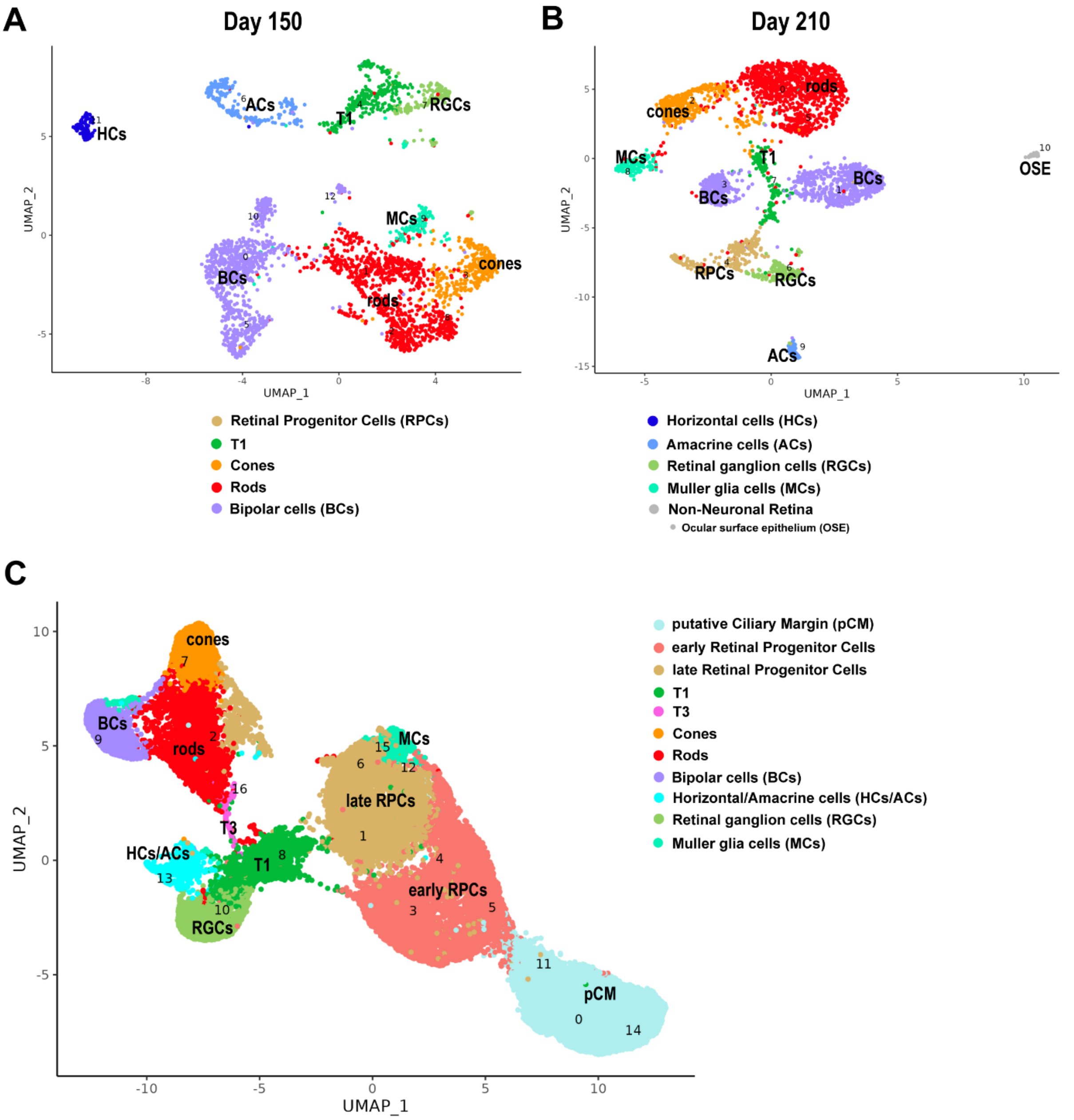

Figure S8: ScATAC-Seq of ROs during late development (day 150 - 210) and integrated ROs UMAP (related to Figures 7, 8 and Table S5). **A, B)** UMAP plots of scATAC-Seq of ROS at day 150 (**A**) and day 210 (**B**). 96 ROs were used for day 150 and day 210. scATAC-Seq was performed once per each time point of RO differentiation. **C)** Integrated scATAC-Seq data of ROs from day 10 to day 210 of differentiation. Cluster identity defined based on gene activity scores, calculated on open regions of the chromatin, of retinal specific cell markers (Table S5).

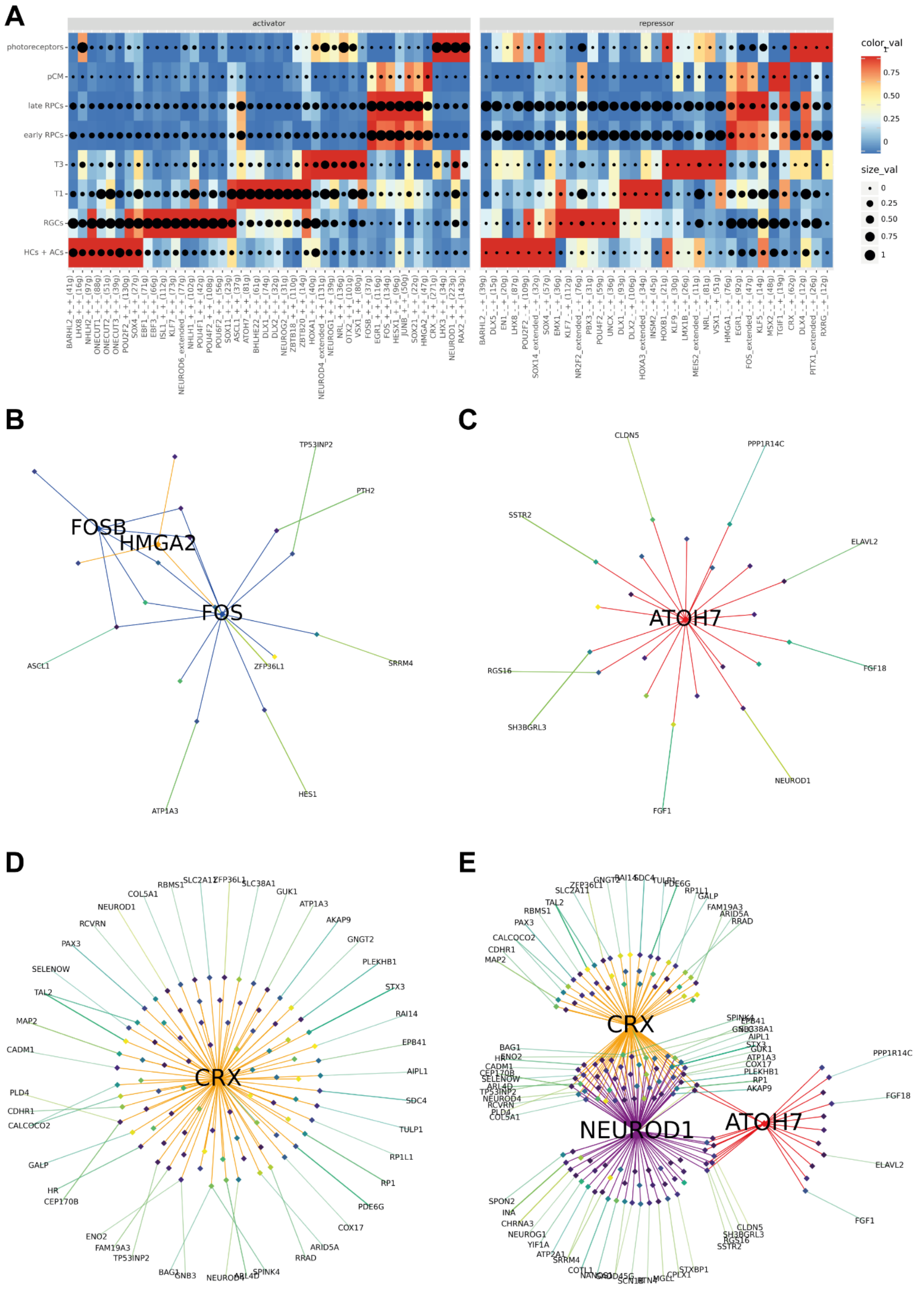

Figure S9: Visualization of SCENIC+ enhancer driven Gene Regulatory Network (eGRNs) analysis (related to Figures 7 and 8). **A**) Shows a heatmap of cell type specific transcription factor (TF) expression within the eRegulon. Colors indicate intensity of expression, and the cell-type specificity (RSS) is represented by the size of the dot. Cell types are ordered according to similarities in gene. eGRN are shown for the: RPC specific TFs, *FOS, FOSB* and *HMGA2* (**B**), T1 specific TF *ATOH7* (**C**),

photoreceptor specific TF CRX (**D**) and a combined T1/Photoreceptor network containing *ATOH7*, *CRX* and *NEUROD1* (**E**). Transcription factors associated with each regulon are shown in the center of the networks in large text. These are linked to candidate enhancer regions (shown with a diamond but not labelled). The diamonds are color coded by differentially accessible region analysis log2fc values for cell types. The candidate enhancer regions are then linked to genes (labelled) within the regulon. The edge color between region and gene is colored by the region to gene correlation score.

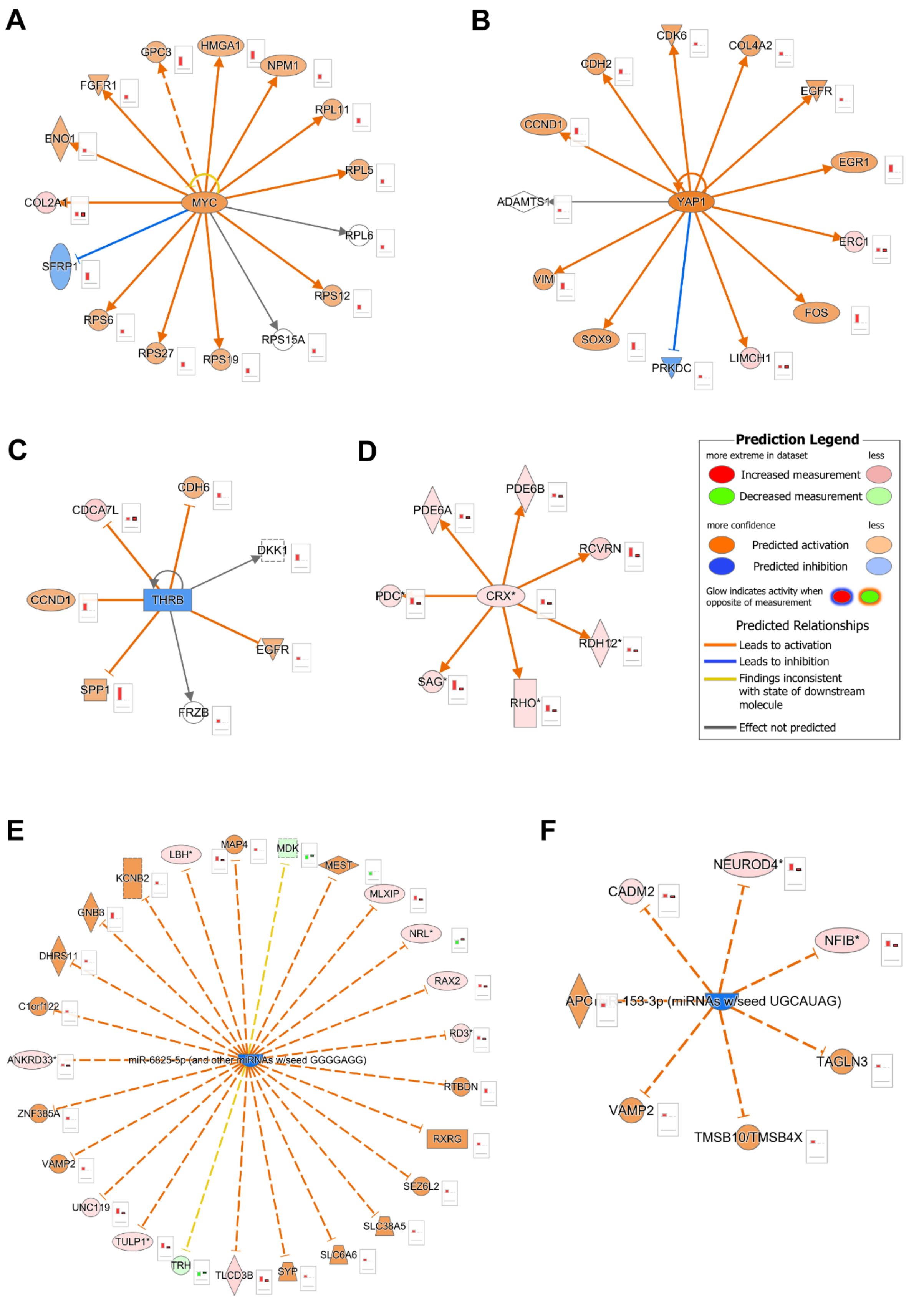

Figure S10: Representative gene regulatory networks in early (A) and late RPCs (B, C), photoreceptors (D, E), and BCs (F) depicting activated and inhibited upstream regulators and their target genes (related to Figures 7, 8 and Table S8). IPA was used to generate the upstream regulatory networks from differentially expressed genes from the scRNA-Seq data and differential accessibility analysis in the scATAC-Seq data (Table S8). These networks indicate predictions of

upstream regulators which might be activated or inhibited to explain observed upregulation/downregulations in the data. The bar plots next to each molecule illustrate the relative expression in the scRNA-Seq (column 1) and scATAC-Seq (column 2) datasets. The network nodes/bar plots colors represent observed upregulation/ increased chromatin accessibility (red), predicted upregulation/increased chromatin accessibility (orange), observed downregulation (green) and predicted downregulation/ decreased chromatin accessibility (blue). The edge color represents the relationships between the molecules; orange = prediction and observation are consistent with activation; blue = prediction and observation are consistent with downregulation; yellow = prediction and observation are inconsistent; and grey relationship between the molecules is not available in the IPA knowledge database. *- indicates duplicates in scATAC-Seq data.

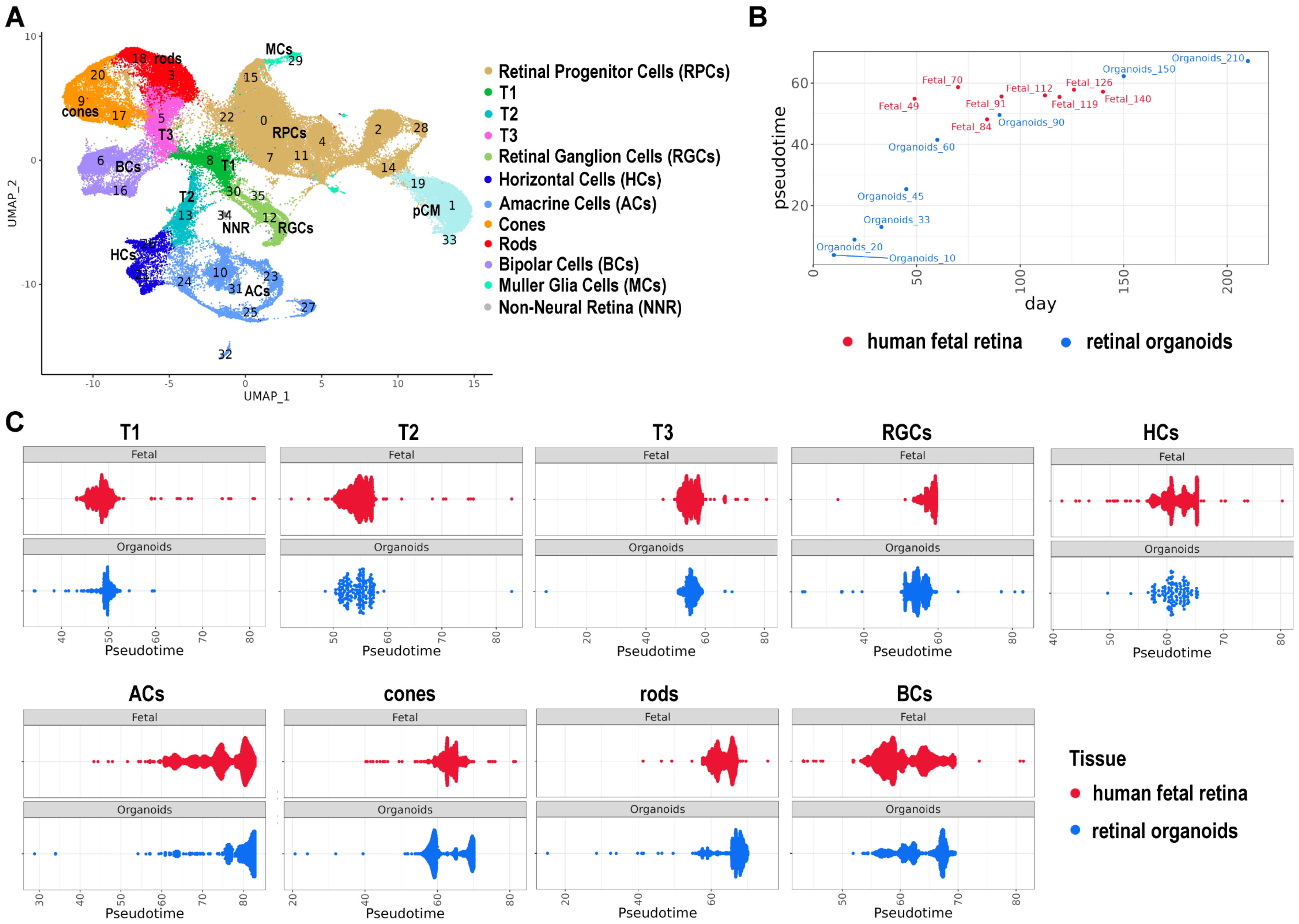
A
B
C
UMAP_1
UMAP_2
Retinal Progenitor Cells (RPCs)
T1
T2
T3
Retinal Ganglion Cells (RGCs)
Horizontal Cells (HCs)
Amacrine Cells (ACs)
Cones
Rods
Bipolar Cells (BCs)
Muller Glia Cells (MCs)
Non-Neural Retina (NNR)
pseudotime
day
human fetal retina
retinal organoids
T1
T2
T3
RGCs
HCs
ACs
cones
rods
BCs
Fetal
Organoids
Pseudotime
Tissue
human fetal retina
retinal organoids

Figure S11: Pseudotime trajectory comparison of chromatin accessibility landscapes of ROs and developing human retina indicates minor differences in the temporal emergence and abundance of the retinal neurons (related to Figures 7, 8 and Table S9). **A)** Integrated UMAP of ROs (day 10 – 210) and human fetal retina (8 – 21 post conception weeks) scATAC-Sec datasets (Table S9). **B)** Grouping of individual developmental stages of ROs (blue) and human fetal retina samples (red) within pseudotime analysis, indicating that RO differentiation ROs closely follows the retinal development *in vivo*. **C)** Pseudotime trajectory analysis of individual cell types demonstrate some differences in T1, RGCs, cone, rod, and BCs emergence between ROs and human fetal retina.

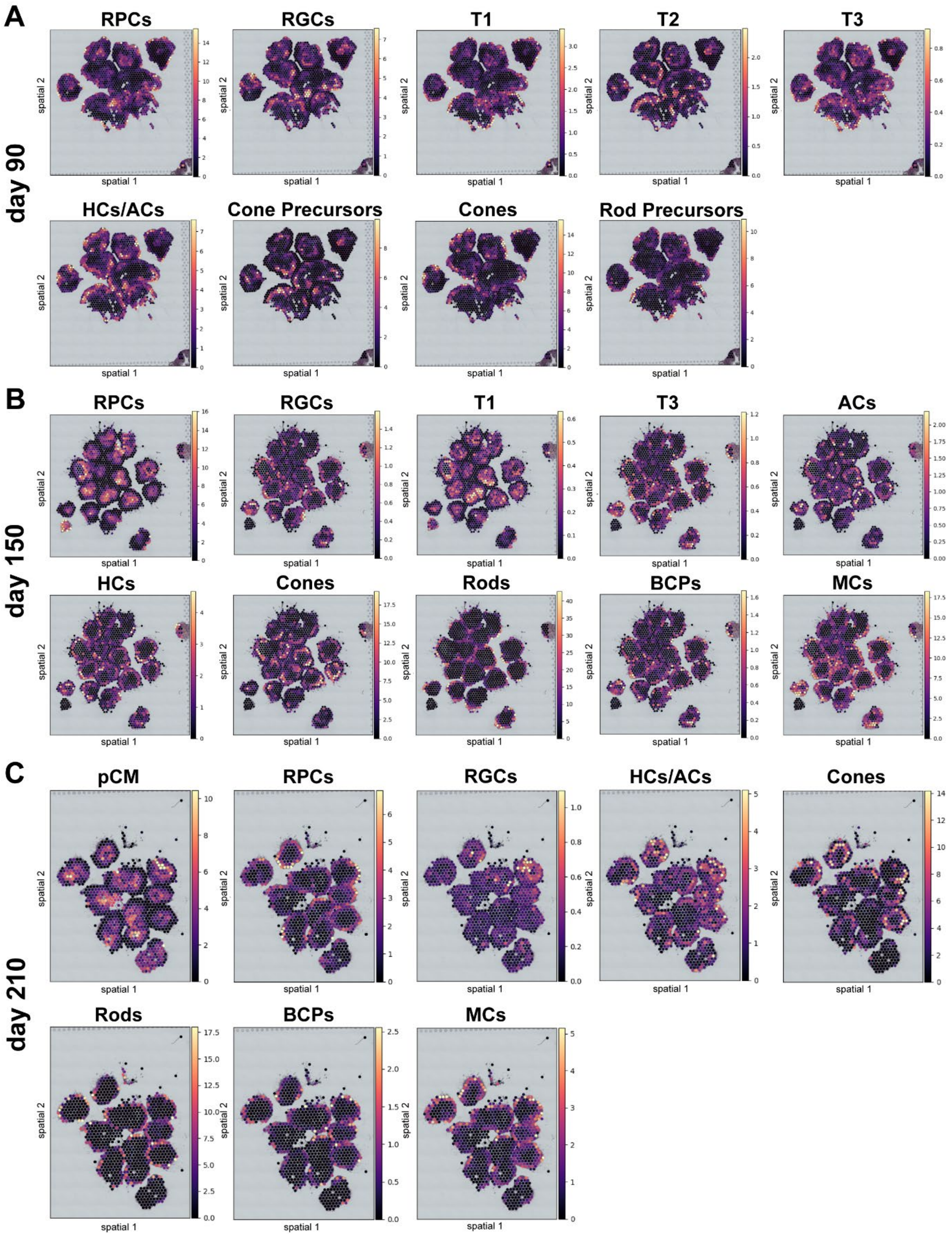

Figure S12: Retinal cell type abundance estimated by Cell2Location is shown for each spatial coordinates for organoids at day 90 (A), day 150 (B), and day 210 (C) (related to Figures 2 and 3).

Table S10 (separate file, related to Figures 1-3)
Media compositions for each stage of RO differentiation.

	Media compositions (concentrations)				
**Product name (Company, Catalogue number)**	**Day 0 - 17**	**Day 19-29**	**Day 30-89**	**Day 90 - 120**	**Day 121 – 200**
**DMEM/F-12: basal media (Thermo Fisher, 11320033)**	500 ml	500 ml	500 ml	500 ml	500 ml
**Knockout Serum Replacement (KOSR) (Thermo Fisher, 10828028)**	20%	–	–	–	–
**Fetal Bovine Serum (FBS) (Thermo Fisher, A5256801)**	–	10%	10%	10%	10%
**Penicillin/Streptomycin (Thermo Fisher, 15140130)**	1%	1%	1%	1%	1%
**B27 Supplement (Thermo Fisher, 17504001)**	2%	2%	2%	2%	2%
**N2 Supplement (Thermo Fisher, 17502001)**	–	–	1%	1%	1%
**Fungizone (Thermo Fisher, 15290018)**	0.25 µg/ml	0.25 µg/ml	0.25 µg/ml	0.25 µg/ml	0.25 µg/ml
**GlutaMAX™ Supplement (Thermo Fisher, 35050087)**	1%	1%	1%	1%	1%
**Non-Essential AminoAcids (Thermo Fisher, 11140050)**	1%	1%	1%	1%	1%
**IGF-1 (Merck, SRP3069)**	5 ng/ml	5 ng/ml	10 ng/ml	10 ng/ml	10 ng/ml
**Taurine (Merck, T8691)**	–	0.1 mM	0.1 mM	0.1 mM	0.1 mM
**T3 (Merck, 709719)**	–	40 ng/ml	40 ng/ml	40 ng/ml	40 ng/ml

**Chemically Defined Lipid Concentrate (Thermo Fisher, 11905031)**	–	–	0.10%	0.10%	0.10%
**Retinoic acid (Merck, R2625)**	–	–	–	0.5 μM	–

Table S12 (separate file, related to Figures 1-3)
Estimated numbers of cells under spot in ST analyses.

sample	apical edge of RO			centre of RO			average
day 10	13	14	20	13	17	15	**15**
day 20	20	13	15	9	13	14	**14**
day 35	17	20	24	9	13	15	**16**
day 45	24	20	22	12	13	16	**18**
day 60	21	18	24	13	9	11	**16**
day 90	22	19	23	9	13	13	**16**
day 150	24	19	21	5	12	11	**15**
day 210	23	18	22	6	9	16	**16**
